# Colored Corn: An Up-Date on Metabolites Extraction, Health Implication, and Potential Use

**DOI:** 10.3390/molecules26010199

**Published:** 2021-01-02

**Authors:** Raffaella Colombo, Lucia Ferron, Adele Papetti

**Affiliations:** 1 Department of Drug Sciences, University of Pavia, Viale Taramelli 12, 27100 Pavia, Italy; raffaella.colombo@unipv.it (R.C.); lucia.ferron01@universitadipavia.it (L.F.); 2 FlaNat Research Italia Srl, Via Giuseppe di Vittorio 1, 20017 Rho (Milano), Italy

**Keywords:** colored corn, purple corn, blue corn, corn waste, anthocyanins, polyphenols, extraction, corn-based food, non-food corn use

## Abstract

Colored (orange, pink, red, purple, and blue) corn strongly attracted attention on its healthy properties mainly due to its anthocyanin and carotenoid composition which is also responsible for its pigmentation. The present review summarized the recent updates on the extraction and chemical characterization of the main plant secondary metabolites present in colored seeds, kernel, cob, husk, and silk. The main approaches used to stabilize the extracts have been discussed as well as their food and non-food uses. Both in vitro and in vivo (animal models) studies on the different effects (antibacterial, antimutagenic, antioxidant, and anti-inflammatory activities, effects on metabolic syndrome, diabetes, glucose and lipidic metabolism, and neuroprotection) of pigmented extracts on animal and human health have been summarized.

## 1. Introduction

One of the most produced cereal worldwide is *Zea mays* ssp. *mays* L. (corn) with about 1.15 billion tons estimated per year in 2018 [1]. Corn is commonly used for feed and food, but also for other different industrial purpose, i.e., pharmaceutical and cosmetic fields.

In the last decades, the scientific research focused the attention on pigmented corn varieties because of the well-known healthy properties of anthocyanins [2,3,4,5,6]. For this reason, breeding technologies have been pointed out to increase the anthocyanin content in uncolored varieties using different approaches, mainly starting from those traditionally cultivated in South and Central America [7]. Purple corn is typical of the Andean Region of South America where it has been used for centuries for the preparation of traditional food and beverages. Recently, it was also considered a good source of natural colorants to be used in textile industry [7] because of its content in anthocyanins; however, it also contained other phenolic compounds showing health-promoting effects. For these reasons, purple varieties attracted the attention of researchers to a greater extent for their potential use in food supplement and cosmetic industries. Anthocyanins are mainly located in pericarp in red and black varieties, while they are concentrated in the aleurone in blue corn [8].

The aim of this review is to summarize the results obtained in the last years about the biological properties tested both in vitro and in vivo, and the food and non-food use of pigmented corn varieties, considering not only kernels and its derived products, but also corn waste, i.e., husk and cob. In addition, different extraction approaches applied to obtain the tested extracts are discussed, as well as the different secondary metabolite profiles in relation to corn color.

## 2. Pigmented Corns and By-Products 

Corn, also known as maize, is an ancient crop, nowadays still cultivated all around the world. Recently, it has been suggested that it was firstly domesticated as staple crop in Honduras around 4300 years ago, based on its discovery in the El Gigante rock shelter in the Honduran highlands [9]. Mythology reported that corn was kept inside a mountain, then divine intervention made it available to humanity by opening the mountain with a thunderbolt. However, the crashing thunder burned the corn, giving rise to the different kernel colors: the black seeds were the outermost, totally burnt, followed by blue, red, yellow and finally white ones, corresponding to the unburned seeds in the center of the mountain [7].

Ancient American corn varieties were reported to be widely pigmented, going from white to black colors. However, after the discovery of America in 1492 and the spread of this crop in Europe, it lost its pigmentation due to the adaptation process to European environmental conditions (longer photoperiod and cold-temperate climate), with only rare exceptions [7].

The corn color is mainly due to the presence of a huge number of secondary metabolites, such as phenolic acids, carotenoids, and flavonoids. The different expression of these pigments imparts to corn tissues different colors, from yellow-orange to dark purple-blue, as well as ivory, and cream colors. Pigments are generally mostly concentrated in the thick pericarp or in the aleurone layers of kernels, and also in corn cobs [10,11,12]. In plants, flavonoids physiological role consists in the recruitment of pollinators and protection of tissues from UV and oxidative stress. Several authors reported that the flavonoid biosynthesis pathways are modulated by two kind of transcription factors, encoded by the bHLH (basic Helix-Loop-Helix) and MYB (MYeloBlastosis) gene families, while WD40 (tryptophan-aspartic acid 40) factor has been exclusively related to anthocyanins structural genes; the interaction of these transcription factors and their target genes resulted in both spatial and temporal biosynthesis of anthocyanins in maize [7,13,14,15,16,17].

Anthocyanins can be accumulated in seeds, corn stalks, cobs, and leaves and their different chemical structure together with their different contents lead to various color shades, from pink to dark blue, which characterized the different pigmented corn varieties.

However, it is well known that maize plant genotype is not the only factor determining corn pigmentation, as different environmental factors, altitude, temperature, and abiotic stress, in addition to maturation stage, could highly affect anthocyanins accumulation in corn [18,19], differently from cultivation practices (for example, potassium fertilization and seeding density) which have less impact, as reported by Jing et al. for eighteen different purple corn cultivars from Perù [20]. The evaluation of the effect of location and genotype, in addition to their interactions, is useful for breeding, as demonstrated by Khampas et al. [21] who selected a distinct genotype grown in a particular zone of Thailand, characterized by high pelargonidin-3-*O*-glucoside and peonidin-3-*O*-glucoside levels and high 2,2-diphenyl-1-picrylhydrazyl (DPPH) radical scavenger capacity. Germination is another factor affecting polyphenols concentration, especially anthocyanins, as demonstrated by the decrease of their amount in purple corn seed, with the exception of peonidin malonylated forms [22].

Generally, purple corn kernels are richer in anthocyanin than blue (4910mg vs. 363 mg/kg dry corn) and red ones [12,18], and this content is very close to that of other food sources, but differently from these other sources it is concentrated in the pericarp and therefore easier extractable, thus it has been pointed out as an extremely valuable source of bioactive pigments [23].

Recently Li et al. [24] investigated the potential use of blue and purple corn by-products obtained from both the dry- and wet-milling processes, showing that, based on the extent of anthocyanins accumulation in the aleurone layers and corn phenotype, highly pigmented coproduct suitable for further utilization could be obtained from these processes. Moreover, several studies reported that high content of pigments in corn were found in the inedible husk, cob, and silk, which ranged from 0.49% and 4.6% of the dry or fresh weight, respectively [11,25,26]. As regards corn silk, it is considered a waste product which could be used as natural colorant, flavoring agent or to produce value-added products such as snack, corn silk tea, or cosmetics. Different silk quality can be achieved according to the harvesting period: In fact, at silking stage it contains the highest amount of polyphenols, differently from what happened at the physiological maturity stage. At immature stage, an intermediate amount was detected [27]. Silk phytochemical composition was also evaluated for four purple waxy corn genotypes (*Zea mays* L. var. *ceratina*) at edible and seed stages by Simla et al. [28]. Corn, cob, and husk were also considered. Silk at both the maturity stages had higher content of anthocyanin and phenolics, as well as antiradical capacity against DPPH and 2,2′-azinobis [3-ethylbenzothiazoline-6-sulfonic acid]-diammonium salt (ABTS) than other ear components. Anyway, cob had a high anthocyanin content independently from the genotype and maturity stage. Considering that silk is used for the preparation of tea/herbal-like beverage, different colored silks (yellow, green, pink, and purple) were compared with six medicinal herbs for their polyphenols and chlorophyll contents. Corn silks at the silking stage were the richest in total polyphenols, with about two- to four-fold higher content than silks at the R4 dough stage, while anthocyanins highly contributed to the color of pink and purple silks and proanthocyanidins were present only in the purple colored variety. Moreover, their polyphenolic content is generally higher than that of *Mentha piperita*, *Ginkgo biloba*, *Thymus serpyllum*, and *Salvia officinalis*, but lower than that of green tea. HPLC-MS analyses indicated the presence of five chlorogenic acids, including 3-caffeoylquinic acid (-CQA), 4-CQA, 5-CQA, and *p*-coumaroylquinic acid, with 3-CQA and 4-CQA present in higher concentration (932.7–1840.8 mg/100 g and 186.9–426.6 mg 3-CQA equivalents/100 g, respectively) and about two-fold content in purple and green silks with respect to yellow ones. Three flavone glycosides were also present, of which maysin and its methoxy analogue are prevalent. A lower chlorophyll a and b content than the medicinal herbs was found, and chlorophyll a was present in higher concentration than b in all the tested samples. Some minerals, i.e., Na, K, Mg, and Ca, were also quantified, with their concentration depending on silk maturation stage and only K level was higher at silking stage than at R4 dough stage. Moreover, as there is not a meaningful difference between corn silks at silking stage and the tested medicinal herbs and considering their polyphenolic profiles, corn silks could be used, similar to tea, as a functional ingredient in food supplements [29].

## 3. Pigmented Maize All Around the World

Anthocyanins rich corn varieties are mainly cultivated in South America, especially in Peru and Bolivia [20]. During these last years, several Central and South American pigmented corn accessions have been deeply investigated for their antocyanins content. 

Several works reported that the 3-*O*-glycosylated (glucoside) forms of cyanidin, pelargonidin, and peonidin, along with their malonylated and succynil derivatives (i.e., cyanidin-3-*O*-(3″-*O*-malonylglucoside), cyanidin-3-*O*-(6″-*O*-malonylglucoside), pelargonidin-3-*O*-malonylglucoside, peonidin-3-*O*-malonylglucoside, cyanidin 3-*O*-(3″,6″-*O*-dimalonylglucoside, cyanidin 3-*O*-(6″-*O*-succinylglucoside), cyanidin 3-*O*-(3″,6″-*O*-malonylsuccinylglucoside), and peonidin 3-*O*-(6″-*O*-succinylglucoside)) are the most common abundant and common compounds detected in these pigmented varieties [30,31]. Among them cianydin-3-*O*-glucoside (C3G) was showed to be mainly expressed in purple and red kernels, differently from its malonylated derivative which was revealed in higher concentration in red/blue and blue varieties [18,32]. Cyanidin-3-*O*-(6″-*O*-disuccinylglucoside) in addition to cyanidin-3-*O*-(6″-*O*-succinylglucoside) was identified as a marker in fifteen blue corn accessions from the northwestern Mexico [33].

Moreover, González–Manzano et al. [34] reported the presence of flavanol-anthocyanin condensed pigment in two different native Mexican purple corn varieties, (cv. Arrocillo and cv. Peruano), in particular the adducts catechin-(4,8)-peonidin-3-glucoside, catechin-(4,8)-cyanidin-3-glucoside, catechin-(4,8)-perlagonidin-3-glucoside, and their malonylated derivatives were identified through NMR analysis. These data were further confirmed by investigating Morado cultivar composition, the most widely diffused purple corn commercialized as a powder: it was shown to contain a particular flavonol-anthocyanin condensed pigment, namely catechin-(4,8)-cyanidin-3,5-*O*-diglucoside, previously found only in wine [35] and some fruits and vegetables [36]. This compound, identified by ^1^H- and ^13^C-NMR using 1D and 2D techniques and by mass spectrometry, was also quantified in seed pericarp, in endosperm and in both parts of three varieties collected in Mexico, together with other cyanidin, peonidin or pelargonidin condensed pigments deriving from a C-C linkage between their C8 and C4 atoms in catechin or epicatechin. It was also noticed that these condensed compounds are not necessarily distributed in relation with the presence of the free anthocyanins, confirmed their naturally presence in some corn variety and not the fact that they formed during corn processing, as previously supposed [34]. Recently, the same molecules were identified in a particular Peruvian cultivar of purple corn (var. PMV-581), both in non-germinated and in germinated seeds [22]. A prevalence of pelargonidin-derived pigments, whose flavanol moiety was represented both by catechin or epicatechin and afzelechin, was highlighted in six different purple corn lines from the landrace Apache Red (AR), accounting for 10.1–18.9% of the total anthocyanin content, differently from Morado purple corn in which they represented 28.3% of the content. Furthermore, AR lines are characterized by the presence of C-glycosyl flavones which could contribute to highlight the health bioactivities and the co-pigmentation, thus leading to further breeding programs to improve natural colorants concentration [37].

According to Collison et al. [18], purple, blue, red/blue, and red kernels of twenty-four different pigmented maize hybrids obtained from Peruvian Morado variety contained 890–3312, up to 540, up to 368, and up to 127 mg of C3G equivalent/g of dry matter, respectively, and a similar composition in anthocyanins was also registered for colored corn cob in addition to the presence of catechin-(4,8)-cyanidin-3,5-diglucoside [11].

Lower anthocyanins concentrations have been detected by spectrophotometric methods in four red and seven blue genotypes from Mexico (3.89–12.09 mg C3G equivalent/100 g of dry matter for red samples and 10.24–34.17 mg C3G equivalent/100 g of dry matter for blue samples) [38]. Moreover, in all pigmented corn varieties, phenolic acids derivatives were also detected, as reported by Montilla et al. [39], who compared both anthocyanins and phenolic acids contents in ten different Bolivian purple corn cultivars. In this work the total content of phenolic acids were quantified using Folin–Ciocalteu assay showing that the sum of soluble and bound phenolics ranges from 311.0 to 817.6 mg GAE/100 g of dry weight, while ferulic acid and *p*-coumaric acid were recognized as the most representative compounds of phenolic fraction present in all the varieties studied by HPLC-Diode Array Detectr (DAD) analysis. On the other hand, anthocyanin fraction was quantified in these ten samples by HPLC-DAD: the registered values were extremely different among the ten studied varieties, ranging from 1.97 and 71.68 mg of C3G/100 g of dry weight. The cultivar Kulli showed the highest anthocyanins content, thus its composition was fully characterized, confirming that cyanidin-, perlagonidin-, and peonidin-glycosylated forms and their malonylate derivatives are the most representative in purple corn.

These data agree with the results reported by Urias–Lugo et al. [40], who compare total phenolic and anthocyanins content of two blue-corn varieties named Chalqueño and Conico with five hybrids, obtained from blue Mexican maize landraces and white varieties characterized by improved agronomic stability and adapted to highlands. Both the native varieties and the hybrids showed a similar composition: with a total anthocyanins content ranging from 646 mg to 1052 mg of C3G equivalent/kg, while the free and bound phenolic fractions content were 1037–1488 and 9064–11987 mg GAE/kg, respectively.

Recently, two open-pollinated varieties deriving from Southwest and Corn Belt blue corn cultivated in different New Mexico locations, namely “Los Lunas High” and “Ohio Blue”, were compared with six landrace accessions from different areas of Arizona and New Mexico for their content in anthocyanins as well as in amino acids, protein, starch, crude fiber, fatty acids, and ash. No significant differences among the tested samples were recorded and therefore they could be suitable for traditional dishes and their cultivation should be encouraged, considering also that the open-pollinated corn varieties were characterized by the presence of cyanidin-3-*O*-disuccynilglucoside [41,42].

Conversely, a high amount, i.e., 915.43 ± 90.07 mg C3G equivalent/kg, was found in a Turkish blue corn flour [43].

Considering European pigmented varieties, rare corn genotypes still display the anthocyanin colorations; among them “Millo Corvo” is an ancient landrace with dark blue/black kernels typically cultivated in Spain’s region of Galicia, and it is particularly rich in anthocyanins (83.45 mg/100g of flours), flavonols (74.21 mg/100g), and phenolic acids (216.63 mg/100g), but it is poor in carotenoids [44]. In this context several Italian ancient pigmented varieties are reported, ranging from orange to dark purple.

Recently the genotype of pigmented “Rostrato Rosso” variety was investigated using an untargeted metabolomics approach based on Ultra-High Performance Liquid Chromatography with ElectroSpray Ionization, coupled to Quadrupole-Time-Of-Flight (UHPLC-ESI/QTOF) mass spectrometry, demonstrating that it is characterized by a particular phytocomplex, rich in both phenolic acids (1149.7 mg/kg), anthocyanins (4399.4 mg/kg), and other flavonols [45]. “Rostrato Rosso” mainly differs from South American pigmented maize for the presence of uncommon anthocyanins forms in pigmented corn, such as malvidin 3-*O*-(6″-acetyl-galactoside), or delphinidin 3-*O*-xyloside, and it is also rich in tyrosol and ɛ-carotene.

“Nero Spinoso” from Camonica valley in Italy, in turn, is an ancient cultivar characterized by the extremely dark black/red coloration mainly due to the high content of phlobaphenes, accumulated in the pericarp layer of seeds thank to a transcription factor belonging to the myb transcription factor gene family (Pericarp 1 (P1) gene). In addition, it contains also flavonoids and phenolic acids and all these peculiarities make this cultivar very interesting as functional food [46].

Finally, Capocchi et al. [47] chemically characterized four genotypes grown in the northern mountain zone of Tuscany whose kernel color ranged from orange to dark red. In red and orange varieties, the pigments accumulated in the pericarp and in the endosperm (carotenoids), respectively. High amount of phlobaphenes were detected in red grains which progressively decreased form dark red to yellow genotypes. High levels of carotenoids were also present in the endosperm of the red varieties with β-carotene being the less abundant, differently from zeaxanthin that is present in considerably higher amount, indicating a possible different biosynthetic pathway in these traditional genotypes. Ascorbic acid was present in a range of 10.33–64.89 mg/g flour with the lower content registered for the yellow variety. Based on these data, the traditional Tuscany corn varieties can be considered good source of nutraceutical molecules.

The cultivation of pigmented corn have been also reported from Asian countries: Khamphasan et al. [48] evaluated the effect of genotype and location, and their interaction, on the anthocyanin composition, total polyphenolic compounds, antioxidant activity, and color parameters of cob and husk from 53 different purple corns from Thailand, giving important information about the way to improve phytochemicals concentration in such corn by-products, which could be recycled for different purposes [48].

In 2016, Russian Research Scientific Institute of Corn published results related to new hybrid varieties with different pigmentation including a variety with grey tops and light yellow sides which had a similar protein content to that of the most conventionally used yellow corn and also a concentration of macro- and microelements very similar to that of a red variety; conversely, white and orange hybrid corn kernels had a higher content of starch and therefore they might be recommended for specific technological purposes. The maximum flavonoid content was registered in orange hybrid corn kernels (80 mg/100 g), while the highest anthocyanin amount in rubin hybrid corn (120 mg/100 g) [49].

**Scheme 1:**
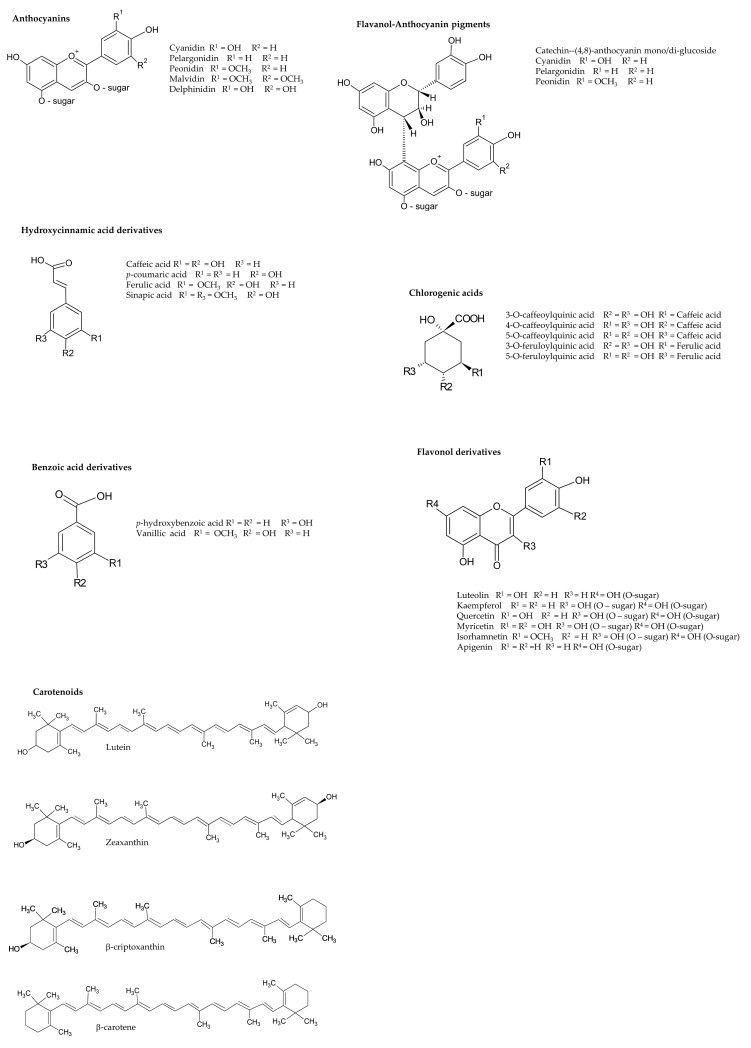
The main classes of compounds considered in this review are listed.

In Scheme 1, the main classes of compounds present in the different pigmented corn varieties are listed.

## 4. Factors Affecting Bioactive Extraction and Stability

Secondary metabolites extraction from plant material is generally influenced by many intrinsic and extrinsic factors, from agricultural practice to pedo-climatic conditions, from extraction method to extraction conditions. Polyphenols and anthocyanins are easily affected by temperature, light exposure, and pH during storage and they tend to degrade into other products; therefore, many techniques over the years have been applied to enhance the stability as well as to optimize the yield of extraction. 

The most common extraction procedures involved the use of aqueous-organic solvent mixture, generally acidified to improve the yield, at a temperature lower than 50–60 °C, for different time [50]. An example is represented by the extraction of anthocyanins from purple corn cob using a food-friendly 50% aqueous ethanol acidified with 6N HCl (0.01%, *v*/*v*) for 45 min and a double washing of the remaining colored insoluble powder after the extract filtration. This extract provided a high level of polyphenols and monomeric anthocyanins without chemically changing which could originate polymeric pigments [51]. However, in 2020 a new approach for water-based anthocyanin recovery from purple corn was investigated. A single-stage, two-stage, three-stage, and two-stage countercurrent water-based extractions resulted in a recovery of 48.6, 68.6, 77.9, and 66.8%, respectively. Moreover, a three-stage process was techno-economic feasible for commercial scale application [52]. 

An improvement in the extraction was generally achieved by ultrasound-assisted extraction by using 95% ethanol with 0.1 M citric acid, and setting the experimental conditions as follows: ultrasonic times 90, power 400 W, and solid:liquid ratio 1:8. This set up was then successfully scaled-up for the extraction of C3G (45% total anthocyanins) and cyanidin-3-*O*-(6″-*O*-malonylglucoside) (40% of total anthocyanins) from purple bran. Lower percentage of pelargonidin-3-*O*-glucoside, pelargonidin-3-*O*-(6″-*O*-malonylglucoside), peonidin-3-*O*-glucoside, and cyanidin-3-*O*-(3″,6″-di-*O*-malonylglucoside) were also obtained [53]. Conversely, a higher content of malonylated (mono- and di-) than non-malonylated cyanidin derivatives was registered for the husk using similar conditions [25]. Different operative conditions were applied to a Mexican red corn cob. Solid:liquid ratio and extraction time highly affected the yield of bioactives which increased from 10 to 30 solid:liquid ratio (*w*:*v*) and from 60 to 120 min of extraction time (from 215.17 ± 33.49 to 527.33 ± 103.79 GAE mg/100 g DW). The use of ethanol 55% allowed the extraction of 5-CQA, *p*-coumaric acid and caffeic acid 4-*O*-hexoside, in addition to three glycosylated apigenin (hexoside, pentosyl hexoside, and 6-C-pentosyl-8-C-hexoside), luteolin-*O*-rutinoside, and scopoletin [54]. Ultrasound-assisted extraction was also the most efficient procedure to extract anthocyanins (1:10 solvent-to-solid ratio, ethanol/water/lactic acid mixture, 80:19:1, for 20 min, 100 W) from Cahuacintle corn and husk. Additionally, an enzymatic-assisted extraction using xylanases, celluclast, and depol, was used since the residual material obtained after the extraction exhibited an intense color. The optimized pH and temperature conditions gave an extract richer in cyanidin-3-O-(6″-O-malonyl)glucoside than the maceration or microwave-assisted extraction methods [55].

Oxidation of polyphenols should be prevented during extraction and the most used method consists in performing this process under N_2_ purging, thus leading to high yield of extraction. In the case of anthocyanins extraction from kernel and cob, applying 0.20 MPa for 15 min under N_2_ purging, 0.492 mg and 1.890 mg C3G equivalents/g of dry kernel and cob, respectively, were extracted at 80 °C by using sample:solvent ratio 1:8 and water:ethanol ratio 1:3 for kernel and 1:1 for cob. This operative condition together with the solvent mixture markedly suitable for anthocyanin polarity promoted the swelling of sample, thus enhancing the contact area between sample and solvent. Other extraction process conditions were studied, but the above reported were identified as the best to avoid both impurity dissolution and anthocyanin degradation which could be at higher temperature, as well as at longer time of extraction [56]. Ohmic heating extraction is another interesting approach that could be used; it was applied to a new purple corn single cross-hybrid from Thailand and the yield of total anthocyanins extraction was compared to that obtained by using other extraction methods based on the use of water as solvent and operating in optimized conditions (conventional extraction applied for 5 min at 70 °C, ultrasound-assisted extraction for 5 min at 80 °C, 37 KHz, and microwave-assisted extraction for 2 min, 2.45 GHz, 700 W). This new approach is based on the rapid change of electrical energy into heat which is able to generate a uniform temperature distribution through the sample solution submitted to the extraction. It has two main advantages: it does not affect the nutritional properties differently from conventional extraction and leads to higher yield in a less time. However, it produces the lowest amount of anthocyanins in comparison with the other methods, and also, the color of the extract is darker, probably due to a quick overheating of cob powder which burnt producing carbon. Microwave-assisted extraction was the best method, and it was scaled-up giving a total anthocyanin yield of 0.25% and producing a dark purple powder arising a deep red colored solution after the dissolution in water [57]. Another interesting extraction method, used to maximize the amount of anthocyanins to be used as dye in food, was a steeping step of the pericarp in sodium metabisulfite generating SO_2_ in presence of lactic acid which facilitates faster SO_2_ sorption. SO_2_ should then promote the pigments extraction by a possible interaction of anthocyanins with HSO_3_^−^ leading to a better diffusion through cell walls and thus to an increased solubility of pigments. Moreover, lactic acid lowered pH of the solution, enhancing anthocyanin extraction and stability. This treatment allowed the extraction of 22.9 ± 0.2 mg C3G monomeric anthocyanin equivalents/g dry pericarp and also of the condensed anthocyanins, differently from the extraction with only water in which the condensed forms were prevalent with only 7.1 ± 0.6 mg C3G/g dry pericarp, or steeping water with 0.2% SO_2_, which extracted only monomeric anthocyanins, but in a lower amount (20.5 ± 1.5 mg C3G/g dry pericarp). Flavonoids concentration did not differ in the different approaches [58]. The foliar part of corn could also be a source of anthocyanins. Its content in a new dark red corn variety was evaluated using a quick, simple and conservative method, namely hyperspectral model, generally used for measuring moisture, nitrogen, and chlorophyll content. To overcome the weak relationship between the anthocyanin content and the hyperspectrum, a sensitive band (685 nm) was selected via multiple linear regression thus enhancing the accuracy and the stability of the model, also when the anthocyanin content was lower than 20 mg/g [59]. 

High-pressure processing for 30–45 min differently affected purple waxy corn kernel polyphenols contents and antioxidant properties depending on the pressure applied. Pressure treatment at 700 MPa was the best condition to have the highest yields in phytochemicals with a color profile overlapping to that of steam-treated kernels. Conversely, lower pressure (from 250 up to 550 MPa) reduced the recovery of polyphenols with a progressively reduction as the pressure decreased. This different action of pressure treatment is probably correlated with the cell rupture and the subsequent release of polyphenols from food materials occurring at the highest pressure level (700 MPa). Moreover, this treatment promoted starch gelatinization with a slower retrogradation than a generic thermal cooking process. Even if high pressure processing is generally not suitable for foods rich in water soluble compounds, the results obtained in this work supported its potential use in the development of new healthy products [60].

Despite the, relatively, easy use of all these methods, the set-up of raw material:solvent ratio, solvent mixture composition, time, and temperature of extraction are time consuming but not so expensive. A great advantage could derive by the use of experimental design. One of the most recently used approach is response surface methodology (RSM). Its application allowed Hwang et al. [61] optimizing solid:solvent ratio, solvent volume, extraction time, and temperature, performing only few experiments to set up the best conditions (at 40 °C for 8 h, with 1:15 in solvent volume, and a 33% solid:liquid ratio) to extract the highest amount of the 3-*O*-glycosidic derivatives of cyanidin, pelargonidin, peonidin, and the malonylglycosidic derivatives of cyanidin and peonidin from a commercially purple corn kernel. In these conditions, the best yield extraction was also obtained for other polyphenols, such as hyrsutrin and its methoxylated derivative, hydroxy benzoic (2,4,6-trihydroxy benzoic, protocatechuic, and vanillic acids) and hydroxycinnamic (ferulic and p-hydroxycinnamic acids) acid derivatives. By using this approach, the same anthocyanins in addition to pelargonidin-3-*O*-(6″-*O*-malonyl)-glucoside, malvidin-3-*O*-(6″-*O*-malonyl-*p*-hydroxybenzoyl)-glucoside, and malvidin-3-*O*-(6″-*O*-dimalonyl)-glucoside were extracted by a purple corn produced in China, without any degradation. RSM was also adopted to optimize the ultrasound-assisted extraction of both anthocyanins and zein (a prolamine protein representing about 40% of total protein content, widely used in different industrial fields for its peculiar characteristics). The results obtained were based on single-factor experiments which considered solid/liquid ratio, solvent extraction composition (ethanol percentage), and power, time, and temperature as ultrasound parameters. The highest yield in anthocyanins (450 mg/kg corn) was obtained using a 1:26 solid (g)/liquid (mL) ratio, 74% ethanol (*v*/*v*) at 70 °C for 90 min, with an ultrasound power of 105 W, after different purification steps consisting in a precipitation with ammonium sulphate precipitation, followed by the separation from zein using a cross-flow ultrafiltration, and a final purification by AB-8 macroporous resins [62]. In another research, a central composite rotatable design (CCRD) was applied to study the effects of two parameters, i.e., temperature and pressure, on many other variables, namely yield of extraction (obtained at each of the three steps applied by sequentially using supercritical carbon dioxide, ethanol, and water), total content of phenolics, flavonoids, and monomeric anthocyanins, anti-DPPH radical capacity and CIE L*a*b* system. Using RSM, the best process conditions for purple corn cob extraction were detected in the use of ethanol both at 65 °C and 450 bar for the extraction of total phenolics (389 mg gallic acid equivalents/g extract) and antiradical activity, and at 45 °C and 420 bar for the extraction of total monomeric anthocyanins (64 mg C3G equivalents/g extract of which 26–38 mg/g were C3G); conversely, the highest yield of extraction for total flavonoids was obtained using water at 50 °C and 400 bar (93.7 mg catechin equivalents/g) [31].

Purple corn cob could be a good and economic source of anthocyanins with its general content higher than kernel, as demonstrated by Lao and Giusti [51]. Thus, it could represent a relatively cheap material for the extraction of natural colorant, even if the cereal texture complexed with other compounds present in cob made more difficult the optimization of the extraction procedure. Muangrat et al. [63] successfully used the central composite face-centered design to develop a RSM predicting the effect of ultrasound amplitude levels and different solvent extraction mixture on total anthocyanin and polyphenol levels. Water-ethanol 1:1, amplitude level of 50%, at 65 °C for 30 min with a sample:solvent ratio of 1:30 were the best conditions identified to extract 27.66 mg and 0.240 mg of gallic acid and C3G equivalents/g dry sample, respectively. The use of ultrasound-assisted extraction has two main advantages: the acceleration of the swelling of cells accompanied by the fragmentation of the tissue matrix whose particles are hydrated by water present in the extraction solvent mixture and thus the penetration of the organic solvent component is facilitated, as well as the intensification of mass transfer by diffusion. Eight main anthocyanins were identified in the extract: cyanidin-3-*O*-(6″-*O*-ethylmalonylglucoside) and petunidin-3-*O*-glucoside in addition to C3G, pelargonidin-3-*O*-glucoside, peonidin-3-*O*-glucoside, and their malonyl derivatives.

All these recent examples of application of RSM to different types of corn and corn waste confirmed the high potential of this approach and its help in the reduction of time and solvent/reagent consumption.

In the last decade the demand of natural colorants increased day by day in order to replace synthetic substances, considered less safe for food purposes, and therefore, stability and color studies became mandatory. Anthocyanins is one of the most studied class of natural colorants together with anthraquinones, betalains, and carotenoids. One of the main issues regarding the use of colored corn extract is its stability. In fact, anthocyanins are red-purple colored in acidic conditions, and following the deprotonation and quinoidal form generation, due to pH increase, they generate a purple-blue color; moreover, metal chelation and co-pigmentation can generate a blue color even in acidic conditions. Other factors influencing the rate of degradation include oxygen, light, temperature, and, also, enzymes. Metal chelation transformed flavylium cations into blue quinoidal structure and these complexes were stabilized by acylation on anthocyanin skeleton [64]. These chelates increased the stability of anthocyanins also during storage and heat treatment even if an excess of metals could degrade these pigments [65]. Anthocyanin chromophores can interact with colorless molecules or intramolecularly with acyl residues on the same anthocyanin by hydrophobic or π-π linkages, thus, generating a blue co-pigment [66]. Recently, a systematic evaluation of the role of acylation in the formation of intramolecular co-pigments was investigated using Al^3+^ and Fe^3+^ as metal ions and performing the experiments at pH 6–7 in the dark and at room temperature for 48 h. The anthocyanins isolated and purified both from a purple corn and a red cabbage extract changed their color more markedly in the case of chelation of Fe^3+^; moreover, at pH 6 these changes were less evident starting from diacylated cyanidin to non-acylated derivatives. An improvement in pigment stability was registered with acylation increasing and among mono- and diacylated derivative the stability was in the order malonic > sinapic > ferulic > *p*-coumaric and *p*-coumaric-sinapic > ferulic-sinapic > sinapic-sinapic, respectively [67]. The addition of ferulic acid at different concentration (4, 8, and 12 mg/mL extract) to a purple corn cob extract obtained by a 24 h maceration of 100 g of powder in 400 mL methanol in the dark at 4 °C determined the co-pigmentation of the antochyanin monoglucoside present in the extract (C3G, peonidin-3-*O*-glucoside, and pelargonidin-3-*O*-glucoside), thus originating the corresponding vinylguaiacol adducts which provided a color-enhancing effect, as demonstrated by CIE L*a*b* color space parameters. Moreover, a stabilizing effect was highlighted when the extract was stored for 90 days at 25 °C in the dark [68]. 

In the past several researchers studied the stability of corn anthocyanins pointing out the greater stability of red/blue with respect to purple/red compounds also during a thermo-alkaline process such as nixtamalization [69]. More recently, the stability of anthocyanin extracts obtained from purple corn pericarp using an accelerated solvent extraction system was evaluated at different pH storage conditions for 12 weeks at 22 °C. The color degradation and chemical stability as the pH increased were confirmed also in this research: in fact, the color differences measured with CIE L*a*b* moved from 0.2 to 3.6 and from 17.7 to 47.5 during 12 weeks of storage, at pH 2 and 6, respectively; moreover, the predicted half-life of total anthocyanins ranged from 44.6 to 60.7 weeks and from 1.8 to 3 weeks, at pH 2 and 6, respectively. Flavanol-anthocyanin condensed forms or pyranoanthocyanins showed degradation kinetics similar to that of monomeric anthocyanins. Based on the obtained acceptable shelf-life data, the authors stated that the pigments extracted from purple corn pericarp can be used as natural colorant in acid beverages [70]. 

In order to stabilize the anthocyanin extracts different approaches based on complexation and co-pigmentation have been evaluated in the last years using Arabic gum or whey proteins or polysaccharides, in addition to metal ions. For example, in 2017 Luna–Vital et al. [71] studied the effect of alginate and zinc on color and chemical stability of purple corn water soluble pericarp extract in a beverage model. Using an experimental design, 13 different samples prepared using a solution of commercially available colorless Jammers cherry flavor (Kool-Aid Invisible^®^, 70.8 g/L) added to extract alone (final concentration 0.5 mg DW/mL) or to extract plus alginate (0.01%) or to extract plus alginate and ZnCl_2_ at different concentrations were tested. Thermal stability was evaluated at 70, 80, and 90 °C up to 6 h and shelf-life at 25 °C in the dark for 12 weeks. Zinc and alginate reduced thermal and shelf-life degradation through the formation of a complex, differently from alginate alone which improved stability only at 70 °C. The Stern–Volmer fluorescence quenching approach was used to assess the complex formation which indicated that the chemical interaction between the fluorophore (anthocyanin extract) and the quencher (ZnCl_2_ or alginate) was due to static quenching, thus slowing the chemical degradation of extract. The results were very promising as both alginate and zinc were used in a lower concentration than the limits which could alter flavor and therefore, could be considered good additives to improve anthocyanin extracts stability. Maltodextrin was also evaluated as carrier in the production of spry-dried colored corn cob extract. Extraction solvents and purification step and drying inlet temperature, in addition to different amount of carrier (2%, 5%, and 10%) were key factors. In particular, inlet temperature highly affected water solubility of the final product and 150 °C coupled with the use of 5% maltodextrin resulted in a 90% of pigment yield, highly soluble with the least color loss [72].

Encapsulation is another approach to improve stability and the use of biopolymer-based hydrogels is essential in the case of food matrices. In the last years, anthocyanin extracts isolated from different matrices have been encapsulated in Ca-alginate hydrogels obtained by incorporation of calcium ions to form the gel matrix [73,74]. In 2018 Guo et al. [75] performed a study evaluating the effect of alginate to pectin ratio, total hydrocolloid concentration, and pH on encapsulation efficiency and stability of anthocyanins from purple corn when exposed to light and temperature. The encapsulation efficiency of hydrogel during manufacturing improved from 26% to 65% by increasing curing bath, but also alginate to pectin ratio and total gum concentration can highly impact this too. Another important result consisted in the improvement of stability of extract in hydrogel spherical particles applying low temperature and high particle weight:solution volume ratio. Finally, the half-life values following a first-order kinetics degradation increased with encapsulation (630 h vs. 58 h of anthocyanin aqueous solution).

Recently, the use of microencapsulation by spray drying has been successfully adopted; in particular, a modified beta-cyclodextrin, hydroxypropyl beta-cyclodextrin, was used as a coating agent able to bind anthocyanins in a more effective way, thus improving the core compounds stability. The thus obtained purple corn powder extract was stable both at 4 °C and 30 °C for 60 days of storage, even if the best storage condition for peonidin-3-*O*-glucoside and pelargonidin-3-*O*-glucoside was 4 °C. At this temperature, a lower increase in the concentration of protocatechuic and hydroxybenzoic acids, deriving from the degradation of anthocyanidin at B-ring level, was registered [76]. Interesting studies were performed on nine different corn extracts obtained by an accelerated solvent extraction system on unique pigmented corn collected by the University of Illinois. The extracts have been added to a colorless cherry flavor beverage model and stored at different temperatures (4 °C, 22 °C, and 32 °C) for 12 weeks. The beverages richest in C3G and condensed anthocyanins maintained the color for a longer time at 32 °C during the entire monitoring time, differently from those containing a high concentration of peonidin derivatives [77].

In Table 1, secondary metabolites of colored corn (kernel, cob, and flour and derived products) in addition to the extraction solvent and method used for their detection are reported.

## 5. Health Benefits

Several in vitro and in vivo studies correlated the healthy properties, i.e., antibacterial, antimutagenic, antioxidant, anti-inflammatory, anticarcinogenic, hypoglycemic, and hypolipidemic, of pigmented corn varieties mainly to their carotenoid, anthocyanin, and polyphenolic content. The different qualitative and quantitative profile can justify the different activity reported in literature, as discussed below.

### 5.1. Antibacterial and Antimutagenic Activities

Polyphenols are known to be antimicrobial agents and this activity against pathogenic bacteria is related to the chemical structure and concentration of each compound and it can also vary dependently on the fact that each molecule is tested alone or in a mixture, and therefore, the composition of the extract can highly affect the activity. Both free and bound phenolic fractions extracted from powdered Peruvian purple corn accession (AREQ-084) were tested on the growth of two probiotic lactic acid bacteria strains, i.e., *Lactobacillus helveticus* and *Bifidobacterium longum*, and of *Helicobacter pylori*, the main responsible pathogen for gastric cancer development [79]. Free phenolic fraction was mainly characterized by the presence of anthocyanins (cyanidin-, peonidin-, pelargonidin-3-*O*-glucoside, and their malonyl derivatives); conversely, bound phenolic fraction was mainly composed of *p*-coumaric acid, ferulic acid, and its derivatives. No effect on the beneficial growth of probiotic bacteria was registered for free phenolic fraction tested in the range 10–50 mg/mL of medium (corresponding to a concentration range of 0.03–0.15 mg C3G equivalents/mL medium) and for bound phenolic fraction tested at 10 mg/mL of medium (corresponding to 0.02 and 0.016 mg phenolic acids and ferulic acid/mL medium, respectively). The same concentrations were tested against the growth of *H. pylori* and no inhibitory activity was registered, differently from the results published for anthocyanins and anthocyanin-rich extracts from cranberry against other Gram-negative bacteria [93]. However, these data were in accordance with previously reported results indicating a similar range of anthocyanin from another source not active on *H. pylori* amount, but active on its virulence factors [94,95]. No relationship between total polyphenol content and antibacterial activity against *Staphylococcus aureus*, *Bacillus subtilis*, *Escherichia coli*, and *Pseudomonas aeruginosa* was registered for different germplasms of corn (white, yellow, and purple) from two different Costa Rica regions, even if a purple variety had high activity against *B. subtilis*, indicating that other secondary metabolites can highly contribute to the biocidal action [96]. 

The antimutagenic activity of eleven genotypes of Mexican pigmented corn (red and blue) was evaluated testing the corresponding flavonoid and anthocyanin extracts using TA100 and TA98 *Salmonella typhimurium* strains and aflatoxin B1 (AFB1) mutagen. No extract was toxic with respect to the bacteria, but flavonoid extracts, rich in ferulic acid, were able to inhibit from 43% to 93% AFB1 mutagenicity with higher capacity to inhibit frameshift mutation in the genoma with respect to base changes mutations. The same trend was registered for the anthocyanin fraction, even if with lower percentage values. In particular blue corn samples, rich in C3G, showed higher efficacy against AFB1 on TA98 than TA100, differently from previously demonstrated for another blue Mexican variety [38,97], but these differences could be attributed to the different methods used to test the antimutagenic activity and also to the different composition in anthocyanins. Furthermore, in the case of flavonoid extracts, the different capacity registered for the tested samples could depend on the different extract composition: in fact, steric and electronic properties of each flavonoid differently affect the capacity to bind bacteria membrane and, thus, penetrate into them [38]. 

### 5.2. Effects on Metabolic Syndrome, Diabetes, Glucose and Lipidic Metabolism

Among the various biological activities reported for polyphenol, and in particular for anthocyanins, the action of the different corn extracts on metabolic syndrome had recently attracted the attention of the researchers. Today, obesity and diabetes are increasing day by day and World Health Organization estimated about 40% of adults and 38 million children are overweight and 1.6 million deaths were estimated to be caused by type-2 diabetes (T2D) in 2016 [98,99]. The search for new antioxidant molecules able to maintain redox status and reduce insulin resistance and inflammation determined by over expression of adipokines, which are signaling factors involved in the regulation of blood pressure, energy and vascular homeostasis, and inflammation, is mandatory. In the last decades, several published research pointed out the anti-diabetic, anti-obesity, and anti-inflammatory potential properties of anthocyanin-based extracts by using in vitro and in vivo model tests and many mechanisms of action have been proposed. Generally, biological models, i.e., cell cultures, offer approaches to study the phytochemicals activity which better correlate with their action in vivo.

Free and bound phenolic fraction obtained by different Peruvian corn accessions were tested in vitro for their capacity to inhibit target enzymes for hyperglycemia and obesity, i.e., α-glucosidase, α-amylase, and lipase activities. Free phenolic fractions generally had a higher dose-dependent activity and darker purple samples highly inhibited lipase and α-glucosidase, but only a mild activity against α-amylase was registered for the same samples, thus leading to promising agents, able to modulate the glycemic levels without abnormal bacterial fermentation of undigested starch in the gut produced by a weak α-amylase activity. A correlation between the anthocyanin content and both α-glucosidase and lipase inhibitory activity was highlighted suggesting a high contribution of these compounds to the registered antihyperglycemic and anti-obesity effects [100]. Similar results were also obtained for a cob extract obtained by a Lombard purple corn variety, namely Moradyn [78].

As purified compounds may exert different biological activities in relation to their chemical structure and the presence of more than one molecule in extracts can exert synergistic or antagonist action, Luna–Vital et al. [80] compared the effect of a purple corn pericarp extract and pure anthocyanins on inflammation, adipogenesis, and insulin-resistance using 3T3-L1 cells as in vitro model and performing the assays both in basal and inflammatory conditions. The extract inhibited the adipocytes differentiation by reducing peroxisome proliferator-activated receptors (PPAR)γ-expression in a dose dependent manner (IC50 0.4 mg/mL and 38.4 μg/mL, respectively) and promoted the reduction of lipid concentration especially in basal conditions, differently from the *pure* single anthocyanins; in fact, a lower triglycerides concentration was detected in presence of IC50 extract, but the same concentration promoted the increase in triglyceride concentration when a post-prandial status was mimicked in the assay evaluating the glucose-dependent lipolysis in 3T3-L1 cells. In addition, the extract reduced the lipogenic and lipolytic enzymes in a dose dependent manner through an interaction with amino acid residues of the catalytic cavity of lipases and different domains of fatty acids synthase, but with IC50 value higher than that of the isolated proanthocyanidin fraction which in turn was higher than those of the isolated anthocyanins. Finally, the extract exerted a general positive effect on insulin-resistant adipocytes in which inflammation was promoted by tumor necrosis factor (TNF)-α, and induced a decrease of leptin and an increase of adiponectin. Intracellular reactive oxygen species (ROS) levels were reduced to basal level both when adipocytes were pre-treated with TNF-α and after treated with extract and when simultaneously treated with TNF-α and extract, differently from pure anthocyanins which generally were more active when the simultaneous approach was used. An improvement of insulin sensitivity was also obtained through glucose transporter type 4 (GLUT4) membrane translocation and the modulation of the phosphorylation pattern of insulin pathway. The same extract and its purified compounds were also active on other two T2D targets, i.e., free fatty acid receptor-1 (FFAR-1) and glucokinase (GK) responsible for the stimulation of pancreatic β-cells to secrete glucose-dependent insulin and the preservation of glucose homeostasis at pancreatic and liver level, respectively. In general, the extract exerted a better activation of FFAR-1 and GK than the pure compounds with IC50 value of 77 μg/mL in INS-1E dual-layer cell culture and of 44 μg/mL in HepG2 cells; therefore, the anthocyanin fraction could be considered a better candidate for ameliorating T2D treatment than pure compounds [81].

The anthocyanin fraction of 20 different purple corn lines deriving from the landrace Apache Red, each with a characteristic polyphenolic profile, was tested using murine lipopolysaccharide (LPS)-activated RAW264.7 macrophages and TNF-α-induced 3T3-L1 adipocytes for testing the anti-diabetic, anti-adipogenic, and anti-inflammatory capacities. The results indicated differences among the genotypes, but generally all samples reduced the production of some target parameters associated to obesity such as the main pro-inflammatory mediators in adipose tissue, i.e., TNF-α, prostaglandin E2, interleukin-6 (IL-6), nitric oxide (NO), LPS, monocyte chemoattractant protein-1 (generally present in high level in obese subjects), leptin, and enhanced the level of adiponectin (an anti-inflammatory mediator). Moreover, the extracts lowered the lipid levels by a cell proliferation inhibition or by a decrease of preadipocyte-adipocyte transition. In this last case, the mechanism of action involved the inhibition of the expression level of PPAR, and it was demonstrated only for some samples as well as the reduction of the expression level of the enzyme fatty acid synthase involved in lipogenesis and therefore indirectly in the control of postprandial blood glucose alteration. Colored pericarp extracts were also able to inhibit α-amylase and a serine aminopeptidase inactivating the glucagon-like peptide-1 with a stronger action than other sources rich in polyphenols [101,102], showing IC50 values ranging from 109.5 to 172.7 μg/mL and from 65.5 to 702.7 μg/mL, respectively. The hypoglycemic effects were also supported by the ability to reduce about 30–60% ROS levels and to restore the glucose uptake (about 30–140%) in TNF-α-induced 3T3-L1 adipocytes. These results supported the potential use of colored corn pigments as functional ingredients for the reduction of the risk of T2D and are fortified by the anti-inflammatory and anti-diabetic actions of quercetin, luteolin, and rutin, and by the anti-adipogenic potential of vanillic and protocatechuic acids generally present in the colored extracts [82].

Another important promising target protein for T2D and obesity treatment is tyrosine phosphatase 1β (PTP1β) which is expressed in different insulin-sensitive tissue. Therefore, some compounds isolated from commercially purple corn kernel were tested using a suitable in vitro kit based on the co-incubation of samples with the substrate N *p*-nitrophenyl phosphate (pNPP). The isolated fraction containing phenolic acids and flavonoids had a moderate PTP1β inhibitory effect (IC50 26.12 μg/mL), even if higher than that of the anthocyanin fraction (IC50 58.20 μg/mL). The most active compounds were ferulic acid, 3-methoxyhirsutrin, pelargonidin-3-*O*-glucoside, and cyanidin-3-*O*-(malonylglucoside); this last one acted with a noncompetitive inhibition of PTP1β [61].

Recently, the antiobesity activity on adipocyte life cycle of a purple corn silk extract was investigated. Chlorogenic acid, quercetin, and naringenin derivatives were indicated as the main responsible for the capacity to reduce significantly the pre-adipocyte proliferation; conversely, the anti-differentiation properties by decreasing the lipid accumulation evidenced for the extract at 0.5 and 1 mg/mL were mainly attributed to quercetin, anthocyanins, and vanillic acid which acted by down-regulating enzymes involved in the adipogenesis and PPARγ expression. Moreover, a moderate lipolysis stimulating effect, which could be a promising therapeutic strategy in the treatment of obesity, was pointed out as demonstrated by the increasing of released glycerol contents in adipocytes, in addition to a moderate apoptosis induction. The overall results obtained in this research indicated purple corn silk as a promising by-product useful in the prevention of obesity, even if in vivo studies are mandatory [26].

The promising results obtained by the above mentioned in vitro studies were generally confirmed by in vivo studies; in fact, purple corn kernel extract provided by the Corn Research Institute in Gangwon Province, Korea, differently affected the metabolic parameters in T2D db/db mice treated for 8 week with 10 or 50 mg dry material/kg: In fact, it significantly decreased blood glucose (52% and 68%, respectively), Hemoglobin A1c (HbA1c) (about 20%), and glucagone (40%) blood levels, but increased the serum insulin (at least of 1.5 fold), C-peptide (at least of 1.4 fold), and adiponectin levels compared to diabetic mice control group. Lower triglyceride levels and higher High-Density Lipoprotein (HDL)-cholesterol levels were also registered in treated groups. The extract determined a suppression of blood glucose level after 30–90 min from glucose load in the oral glucose tolerance test and prevented islet destruction as demonstrated by histological examination. Finally, it activated 5’-adenosine monophosphate-activated protein kinase (AMPK), thus reducing the hepatic glucose production and over expressing mRNA of GLUT4, thus increasing the flux of glucose into skeletal muscle. These results confirmed that purple corn extract is a good candidate for hyperglycemia and hyperlipidemia control in T2D [79].

The effect on metabolic syndrome was also demonstrated in vivo, as shown in the study of Bhaswant et al. [103]. The administration to mail Wistar rats of a high-carbohydrate or high-fat diets, corresponding to 1.8 g/day (9.1 mg anthocyanins/kg/day) or 1.5 g/day of purple corn flour (7.4 mg anthocyanins/kg/day) showed an important impact on metabolic syndrome; in fact, glucose tolerance improved, systolic blood pressure, total body fat mass, and visceral adiposity index were reduced. In animals fed with a high-fat diet, alkaline phosphatase, alanine transaminase, and aspartate transaminase were reduced, even if these parameters did not be normalized, and plasma triacylglycerols and total cholesterol levels significantly decreased. A lower anthocyanins concentration (2.92 mg/kg) from blue corn extract was added both to high-sucrose and high-cholesterol–high-sucrose diets of Wistar rats. The obtained results were again promising as higher high-density lipoprotein cholesterol and lower systolic blood pressure, total cholesterol, serum triglycerides, and epididymal adipose tissue weight were registered in the treated animal group in respect to the control [104].

Purple corn anthocyanins also positively affected the lipid metabolism in lactating rats supplemented with chia oil rich in α-linolenic acid (ALA), in particular they could decrease gene expression of Sterol Regulatory Element-Binding transcription factor 1 (SREBP-1c) in the liver, differently from ALA which would increase the expression of PPAR-α. These actions alter hepatic Δ5 desaturase gene expression only apparently because the purple corn extract protect desaturases activity, which is reduced by high level of ALA with a mechanism not yet totally elucidated [105].

In 2020, the potential mechanisms of interaction of blue corn anthocyanins with target cell signaling proteins involved in inflammation, insulin resistance, oxidative stress, glucose and lipid metabolism, and therefore, in the development of diabetes, was predicted using in silico approach. 312 different interactions between compounds and proteins were detected and the main important occurred between C3G and 11β-hydroxysteroid dehydrogenase (-HSD), glutamine fructose-6-phosphate amidotransferase (GFAT), PPARγ, or petunidin-3-*O*-glucoside, 11β-HS and protein tyrosine phosphatase (PTP) or delphinidin 3-*O*-glucoside, and 11β-HSD, GFAT, PTP, and receptor tyrosine kinases. These molecular docking results gave an important contribution to the search for new molecules with anti-diabetic effect and thanks to these predictions new pathways by which anthocyanins exert their effects can be studied [86].

Diabetic neuropathy is one of the most common complication of diabetes, which can be caused by an increase in oxidative stress status, in advanced end glycation products formation, and aldose reductase activity. A 50% hydroalcoholic extract constituted of *Zingiber officinale* rhizomes and purple *Zea mays* seeds administered to streptozotocin-diabetic rats improved neuropathy with a mechanism not related to the reduction of blood glucose. Moreover, this extract reduced myelin degradation, thus increasing myelinated nerve fiber density, which is crucial for maintaining postural stability in rats subjected to chronic constriction at sciatic nerve, and decreased neuropathic pain by a reduction in the stimulation of pain fiber as a consequence of a reduced oxidative stress [106].

### 5.3. Anti-Inflammatory Properties

Cyclooxygenase-2 (COX-2) and inducible NO synthase (iNOS) are the key factors in the inflammation process and their inhibition could be beneficial. Proanthocyanidin (mainly with degree of polymerization < 10) fraction of purple and red corn pericarp had the capacity to inhibit over 89% COX-2 when tested at the concentration of 1 mg/mL without any effect on COX-1, the key enzyme in homeostatic regulation. The same compounds and C3G present in purple corn were also dose-dependent (0.1–1 mg/mL) non-competitive inhibitors of iNOS [85]. As recently reported by Zhang et al. [107], anthocyanins present in purple and red corn also ameliorated the paracrine interplay between adipocytes and macrophages, reducing the production of TNF-α and Monocyte Chemoattractant Protein-1 (MCP-1) in both macrophages and adipocytes mono-cultures, and inhibiting nuclear factor kappa-light-chain-enhancer of activated B cells (NF-κB) and c-Jun N-terminal Kinase (JNK) pathways activation. 

### 5.4. Effect on Oxidative Stress

An imbalance between the generation of free radicals (ROS and reactive nitrogen species (RNS)) and the antioxidant defense systems caused oxidative stress. Both enzymatic and non-enzymatic systems can contribute to the defense of cell structures and many different molecules other than micronutrients and minerals are known to prevent radical formation or to scavenge the produced radicals. Oxidative stress and inflammation are associated to many diseases, and also to obesity, a complex disorder co-responsible of the increased morbidity and mortality in the last decades. A purple corn extract containing 52.02% C3G and its malonyl derivative (27.33%), 13.58% peonidin-3-glucoside, and its malonyl derivative (7.07%) decreased oxidative stress and inflammatory status when administered to C57BL/6 mice (200 mg/kg) fed with a high-fat diet (HFD) for 12 weeks. In fact, a reduced production of TNF-α, IL-6, iNOS, and NF-κB in addition to a lower extent of hepatic and serum lipid peroxidation was registered, as demonstrated by a meaningful reduction of serum and hepatic triglyceride (TG), total cholesterol (TC), low density lipoprotein cholesterol (LDL-C), and malondialdehyde (MDA) levels respect to HFD control group. This extract also increased the total serum superoxide dismutase (SOD) and glutathione peroxidase (GPx) activities and reduced TC levels differently from black soybean extract which mainly contain petunidin-3-*O*-glucoside (69.89%), delphinidin-3-*O*-glucoside (27.17%), and a very low amount of C3G, pelargonidin-3-*O*-glucoside, and peonidin-3-*O*-glucoside, probably due to the different composition in anthocyanins. Moreover, fatty acid decomposition is promoted and accelerated as evidenced by the increased fecal butyric acid level. Considering the overall effect of purple corn extract on mice, it could be considered very promising as agent preventing obesity via oxidative stress reduction [89]. Nixtamal treatment of blue hybrid corn increased SOD1 gene expression and total liver antioxidant capacity in rats fed a high-fat diet in respect to raw corn, thus statistically reducing liver inflammation [108].

The enhancing effects on the activity of SOD by anthocyanins have been known for over a decade; in fact, this action and the one on both mRNA expression and blood and urine oxidative status markers were monitored in sheep treated for 14 days with a purple corn anthocyanin-rich Japanese extract (consisting of 78% anthocyanins, 20% ethanol, and 2% citric acid, and added to animal diet at 0.5% anthocyanin concentration) in a crossover design [109]. The monitoring of urine 8-hydroxy-2′-deoxyguanosine (8-OHdG) and plasma MDA levels (markers of DNA and lipid oxidation, respectively) indicated that both treated and control animals did not undergo severe oxidative stress during the study, but a significant increase in plasma SOD activity was registered for the treated group, suggesting a positive mitigation activity of oxidative stress through the extract action on SOD activity. No variation in the main non-enzymatic antioxidants concentrations, i.e., total antioxidant capacity and total glutathione, was registered, differently from previous other researches testing the action of anthocyanins on these markers in monogastric animals [110], probably due to the poor absorption of the same anthocyanins by sheep. These results are promising, even if the researchers concluded that further studies are needed to understand the capacity of purple corn extract to counteract oxidative stress. Purple corn silage also increased blood SOD concentration (9,333 vs. 8,467 U/mL) together with the milk production (31.7 vs. 29.2 kg/day) in lactating cows fed with rations containing 32% dry matter extract, ad libitum for 12 weeks, even if the anthocyanins concentration significantly decreased after 4 weeks of storage (70 mg/kg vs. 20 mg/kg dry matter) probably due to their degradation [111]. This positive effect on lactation was confirmed by Tian et al. [112] who demonstrated that the consumption of anthocyanin-rich purple corn stover silage by dairy goats for 7 days, after 14 days of adaptation to the new diet, increased not only the plasma antioxidant capacity (SOD activity), but also the levels of antioxidant gene expression (SOD2, Glutathione Peroxidase 1 (GPX1), and Glutathione Peroxidase 2 (GPX2)) while decreased mammary gland TNF mRNA expression in respect to normal diet goats. Moreover, a positive correlation between total antioxidant capacity and pelargonidin, SOD and peonidin, and SOD and malvidin-3-*O*-glucoside present in the extract was pointed out, demonstrating that anthocyanins can be transferred to goat milk [113]. 

### 5.5. Effect on Memory and Neuroprotection

The first report about the effect of the anthocyanin fraction present in blue corn tortillas on learning and memory capability was published by Aguirre López et al. [114]. In fact, adult rats fed with 6 g/day of blue corn tortillas for 38 days showed an improved learning and short-and long-term memory when assessed using Barnes’s labyrinth at the end of the feeding period, differently from rats fed with white corn tortillas. In the same year, an extract obtained from purple waxy corn cob and pandan leaves was tested for its potential neuroprotective effect and memory enhancer in menopausal-model Wistar rats. For 28 days, rats were orally given 20, 40, and 80 mg/kg and non-spatial memory was evaluated using object recognition test every 7 days. In addition, oxidative stress status, acethylcolinesterase (AChE) inhibition effect, extracellular signal-regulated kinase 1/2 (ERK1/2) expression in the prefrontal cortex, and neuron density were evaluated at the end of the monitoring period. The lowest dose extract was able to reduce AChE activity, while an increasing in the ERK1/2 expression was evident for the dose 40 mg/kg; neuron density and non-spatial memory improved at all the tested concentrations as well as the reduction of oxidative stress status [115]. The same extract, tested using the same animal model, also improved spatial memory through the suppression of AChE activity and neuron density in dentate gyrus of hippocampus through a better expression of ERK1/2. The registered effects globally enhanced the cognitive function as well as donezepil, a drug usually used in the treatment of memory impairment; therefore, this extract could be considered a good supplement at low cost, taking also into account that its LD50 was more than 2000 mg/kg body weight [116].

### 5.6. Antiproliferative Effect

Blue corn and tortilla extracts obtained using ethanol acidified with 1M citric acid were tested for their antiproliferative effects against hepatocellular carcinoma (HepG2), lung carcinoma (H-460), cervix adenocarcinoma (Hela), mammary adenocarcinoma (MCF-7), and prostate cancer androgen dependent (PC-3) using MTT assay. An antiproliferative effect was registered for all cell lines at 1000 μg blue corn or tortilla extract/mL, with Hela cells being the most sensitive, suggesting that even if monomeric anthocyanin concentration can be reduced following the nixtamalization process in tortillas production, other active polymeric structures can be generated; this effect was also supported by the increase in antioxidant activity and by the same IC50 value of corn and tortilla extracts for the lipid peroxidation inhibition [87]. Purple and red corn extracts were also tested for their anti-proliferative effect on HCT-116 and HT-29 human colorectal cancer cells. Both the extracts suppressed angiogenesis and promoted apoptosis in the tested cell lines, as demonstrated by the reduction of Vascular Endothelial Growth Factor (VEGF) expression and angiopoietin receptor Tie-2 marker in HCT-116 and by their impact on markers of apoptosis (B-cell lymphoma 2 associated X protein ( Bcl2 associated X*BAX), B-cell lymphoma 2 (Bcl-2), cytochrome c, TRAILR2/D5) in HT-29 cells. Computational docking studies pointed out a negative value for free energies of bindings between anthocyanin fractions and receptor and non-receptor tyrosine kinases, thus indicating a spontaneous reaction and therefore the capability to suppress angiogenesis [84].

Recently, new nanoparticles obtained using purple waxy corn and blue butterfly pea petals extract were tested for their potential use in the treatment of cholangiocarcinoma (CCA), one of the poorest prognosis cancer, also resistant to the main chemotherapy drugs. A dose- and time-dependent anti-proliferative action of the new formulation was registered both in CCA cell line and in in a gemcitabine-resistant CCA cell line (KKU214GemR). Moreover, the efficacy of gemcitabine against KKU214GemR cells was enhanced by the co-treatment with low doses of extract, indicating a possible cytotoxic effect carried out by the downregulation of forkhead box protein (FOXM1), NF-κB, Bcl-2, and by induction of mitochondrial superoxide production [117].

### 5.7. Other Effects

An anthocyanin-enriched purple corn extract was tested against the development of orofacial allodynia induced in male rats by the injection of complete Freund’s adjuvant, which caused temporomandibular joint (TMJ) inflammation. This extract, differently from the yellow corn extract, was able to prevent allodynia with efficacy very similar to that of acetyl salicylic acid when added to water starting 11 days before the beginning of the experiments and up to the end 3 days later, and only a partial additive effect was registered following the co-administration of the two agents. Purple corn extract possibly involved the reduction of trigeminal macrophage infiltration and the shift of microglia cell polarization to an anti-inflammatory phenotype, as demonstrated by a reduction in the expression of pro-inflammatory molecules, an increase in the production of anti-inflammatory mediators, and thinner and longer microglia cell processes which significantly contribute to neuronal pain [118].

Another interesting potential use of purple corn extract was tested in Wistar rat; a 50% extract cream was used to protect animals from the deleterious action of UV-B. The obtained results were interesting as when mice were exposed with UV-B (840 mJ/cm^2^) for 4 weeks, a lower increase in matrix metalloproteinase-1 (MMP-1) level (an enzyme responsible for collagen breakdown in photoaging) and a reduced decrease of collagen amount in dermis were registered in mice group using purple corn cream with respect to the control group using a placebo cream [119].

A mixture of purple waxy corn cob and blue petal of *Clitoria ternatea* were incapsulated in niosome and incorporated into a mucoadhesive gel and tested for its capacity to act as a topical oral wound healing in vitro and in vivo. Anthocyanins created intra- and inter-molecular interactions generating nano-sized clusters of crystalline-like aggregates in this system, thus improving permeation. Niosome gel (1% and 10%) promoted the production of collagen in human gingival fibroblasts in in vitro test and was able to totally heal buccal wounds in Wistar rats after 5 days, possibly due to the improved mucosal permeability in addition to an anti-inflammatory effect exerted by the topic application [120].

The cardioprotective activity of an extract obtained by a new variety of corn rich in anthocyanin (a hybrid carrying the B1 and Pl1 alleles, which confer anthocyanin pigmentation) was evaluated in wild-type C57BL/6J mice, in which an intraperitoneal injection of 15 mg/kg body weight of doxorubicin (DOX), a chemotherapeutic agent commonly used in the treatment of blood, breast, ovarian, and lung cancers, induced cardiotoxicity. Mice were fed with a diet based on 29% of corn (0.21 ± 0.1 mg anthocyanin/g powder) both for 3 weeks before and for 74 days after the induction of cardiotoxicity. A significant reduction of heart histopathological alterations in addition to a longer survival of treated animals than mice fed with normal diet was evident, confirming the previous results obtained in vitro on murine HL-1 cardiomyocytes pre-treated with C3G standard or purple corn extract, even if these treatments did not affect the cytotoxic effect of DOX on human cancer cell lines [121].

An aqueous extract of purple corn was also demonstrated to possess aphrodisiac activity when administered to male rats in the range 25–75 mg/kg, by controlling ejaculation and by acting at the level of the central nervous system that control the brain and the spinal cord. In fact, the consumption of this extract significantly affected the arousal and execution of sexual behavior without significant influences on the ambulatory behavior and promoted an increase in the number of discharges of the ejaculatory motor [122].

Finally, an interesting work pointed out the effect of 5% purple corn extract supplementation of rainbow trout diet for eight weeks. A higher n-3 and n-6 polyunsaturated fatty acids contents in fish body was registered as well as a lower adiposity; moreover, a higher gpx1 expression in erythrocytes and plasma total antioxidant capacity (TAC) indicated a potential effect against lipid peroxidation also in fish [123].

In Table 2, a summary of the in vitro and in vivo studies correlated with the health benefits discussed in the above section are reported.

## 6. Colored Corn-Based Foods and Technological Aspects

Technological processes and thermal treatment applied in the different cooking methods highly affected colored corn-based food. In particular, thermal treatment caused significant decreases in polyphenolic compounds concentration and antioxidant activity; moreover, sometimes it altered the polyphenolic composition. The type of cooking is also a key factor because generally boiling caused a more sensitive decrease in corn antioxidants than steam cooking, and anthocyanins as well as other phenolic compounds are lost into the cooking water which could be considered a valuable co-product. A greater loss of healthy compounds was registered for kernels removed from the cob prior cooking, differently from kernels cooked on the ear [83]. 

The use of corn flour in the design of gluten-free products has been recently increased thanks to several studies on the doughs rheological behavior and on the parameters affecting such doughs (i.e., particle size, temperature, water absorption, and shear rate), which are fundamental for the selection of the technological conditions. Corn variety and milling conditions highly affected chemical characteristics of flours and rheological properties of doughs; in fact, the particle size of yellow and purple corn flours were generally smaller than those obtained for white flour when the same milling process was applied and the water absorption as well as the damage to starch present in corn increased. Moreover, elastic behavior was predominant in relation to viscous component and the viscoelastic moduli values decreased. The rheological characterization results suggested that dough properties were strongly related to processing and raw corn characteristics [124]. Moreover, purple genotypes had higher gelatinization enthalpy than non-purple ones due to the fact that anthocyanins increased starch granules fragility during cooking (these genotypes had a lower amylose content in relation to non-purple), as demonstrated by Sebastián Mansilla et al. [125] for purple maize flour from Argentina. In addition, a negative correlation between anthocyanins content and the amylopectin retrogradation enthalpy was evident, probably due to interactions formed after starch gelatinization. Interesting results were obtained by Camelo–Méndez et al. [126] who evaluated the addition of different amount of blue corn flour to composite pasta containing chickpea and unripe plantain in order to develop a gluten-free product. The final product was characterized by a dark color and a high polyphenols content (especially pasta containing 75% blue flour) also after extrusion (20% lost) and cooking (30% lost), and by higher adhesiveness and lower hardness and chewiness than the corresponding white corn-based pasta. The extrusion process was responsible for a lower peak viscosity than the corresponding raw product, more marked for blue flour pasta than the corresponding white, due to the disorganization of starch during the process; however, a second peak viscosity was registered upon cooling for blue pasta indicating a re-arrangement of starch components which could contribute to the retention of polyphenols. Another example of blue corn use in extruded snacks was that relative the preparation containing orange bagasse (the main waste deriving from juice preparation) as a fiber source. Orange bagasse also made hard the extrudates (as showed by the reduction of expansion index) which were characterized by a high numbers per area and small size pores. In addition, starch lost its semicrystalline structure as a consequence of high temperature and mechanical shearing, as showed by infrared spectra and X-ray diffraction patterns [127]. The potential use of purple corn flours in designing of gluten-free eggless muffins without gum addition was demonstrated as well as of white and yellow accessions. The low breakdown viscosity indicated a thermal stability for all the tested accession, even if they differed for physicochemical, antioxidant, and pasting properties. A positive correlation between cohesiveness and chewiness was registered; conversely, a negative correlation with paste viscosities was undelighted. Sensory analysis of muffins prepared from purple corn revealed that they were not so acceptable, differently from those prepared from yellow accessions. These preparations are promising, especially considering the fact that they are eggless and their use could be helpful to counteract the increase in incidences of egg allergies [21].

Common Latin American dishes are based on masa, obtained by the nixtamalization process consisting of an alkali thermal treatment of corn to obtain a dough or a flour (nixtamalized corn flour) after a drying process which reduces water content to 8–10%. This process highly affects thermal, physicochemical, sensory, and also nutritional properties of nixtamalized products [128]. In fact, a high protein content, calcium, and fiber characterized tortillas, tortillas chips, and other Mexican food produced using masa and nixtamalized flour. Moreover, corn wet milling is easier because of the germ and endosperm absorption of water and calcium and gums are released by pericarp and germ during washing phase of the process, thus reducing the cohesive and adhesive properties of the masa [129,130]. Significant differences in the physical properties of different blue corn varieties were registered during nixtamalization process, such as shorter cooking times for the softer kernels. In such conditions, consistency and stickiness masa and hardness and extensibility of tortillas overlap with those of the hard (flint and dent) varieties. Moreover, a reduction in yellow and reddish tones and an enhance in green tones was registered following nixtamalization and all tested flour varieties showed similar gelatinization temperatures. Conversely, dent and flint varieties of flours had the greatest enthalpy values due to a more organized kernel structure. Rheological and thermal parameters indicated that generally the Southwest floury varieties are more suitable for tortilla production [131]. The traditional nixtamalization and lime-cooking extrusion process more markedly decreased carotenoids concentration and the oxygen radical absorbance capacity (ORAC) in red Mexican pigmented corns than in yellow varieties. Only lutein concentration was less affected in traditional and extruded tortillas [132]. Different nutritional value and bioactive compounds concentrations were registered for artisan handmade and commercial/industrial tortillas prepared using white and blue corn. Commercially produced white tortillas had a lower antioxidant capacity, fiber, calcium, and free polyphenols content than blue handmade ones; in particular, blue handmade tortillas had 4.5-fold ferulic acid content compared with white commercially produced. Therefore, artisan fresh tortillas had superior nutritional-nutraceutical properties compared to white commercial products [133]. Huitlacoche (Ustilago maydis DC Corda) is a fungus traditionally consumed in nixtamalized products as it affected corn for two months a year, generating smut on ear, stem, and leaves, and it is a good source of nutraceuticals as well as protein, fiber, and water. Its addition to nixtamalized blue corn flours at different percentages (from 3% to 18%) significantly affected color, water-absorption, adhesiveness, and, in general, the rheological properties of flour, as recently demonstrated by Amador–Rodríguez et al. [134]. In particular, an addition of 3–9% exhibited good machinability in an industrial tortilladora machine, and therefore, huitlacoche could be used as a healthier ingredient in the production of food.

Blue corn varieties are well acceptable when used in the production of popcorn, chips, and flour-based confectionary not only for the attractive color, but also because they have important nutritional and healthy properties due to their high anthocyanin and fiber contents, as confirmed in a recent investigation on Turkey varieties. In fact, 61.12 °C, 64.35 °C, and 75.65 °C were the temperature values registered for the gelatinization onset, peak, and end, respectively. Considering their nutritional composition, the mean contents of moisture, lipid (with oleic, linoleic, palmitic, and stearic the main fatty acids), total protein, and crude fiber were 9.39%, 4.30%, 13.13%, and 2.68%, respectively. Total starch and resistant starch were 63.94% and 8.89%, respectively [43].

Another interesting use was in the production of polvorones, a typical energetic and nutritional Mexican bakery product. This food had better color, flavor, and acceptability than that produced using wheat flour. In addition, the presence of anthocyanins highlighted healthy beneficial properties as the only degradation registered during the transformation of raw flour are the de-acylation of some acylated anthocyanins [88]. 

A combination of purple corn and rice flours as substitute of wheat flour in bakery product was also investigated. A ratio of 71.1:28.9 ensured the highest anthocyanin concentration and antiradical activity, and the best texture score of purple corn cake containing corn silk. Its acidification with 3% fumaric acid preserved the color and polyphenols degradation and therefore this product could be considered a high nutritional and healthy food [135].

As regards the production of liquid food, a modified “Sendechó” beverage was proposed by Flores–Calderón et al. [136] in 2017 who added hops and brewer’s yeast to guajillo chili and blue corn malt (the traditional ingredients), thus obtaining a beverage with the same characteristics of Sendechó in addition to that of corn beer due to an ale fermentation process. Different levels of hops, guajillo, and the addition of caramel malt to blue corn malt were used to prepare eight blue corn malt beers tested for their total acidity, bitterness units, total reducing sugars, alcohol, cis- and trans-iso-α-acids concentration, total anthocyanins and polyphenols, as well as their scavenging activity against ABTS radical cation and DPPH. All these parameters were monitoring during three different stages of the process, i.e., boiling-wort, fermentation—green beer, and maturation—mature beer, and the results indicated that all, with the exception of pH, total acidity, and alcohol content, were affected especially in mature beer. In fact, a marked decrease in polyphenolic concentration and antioxidant activity was registered. However, low-alcohol beers with pH, total acidity, bitterness units, iso-α-acids level, and total reducing sugars similar to barley beers were obtained. Beers based on 85% corn malt and 15% caramel malt showed the highest anthocyanins content and scavenging activity against ABTS radical. Peruvian Andean corn variety, typical of a limited Peruvian zones, is used to prepare a drink, namely chicha morada, containing an extract of the purple pigments, fruits, such as pineapple, quince, apple, and peach, which conferred flavors, and a starch source (potato, corn, or sweet potato). If a cooking process is applied and dried or fresh fruits are added, mazamorra morada, a dessert, is obtained. This particular variety had important nutritional and health effects and its flour can also be used in other foods, (pasta, bread, and cakes), providing color and flavor to the processed products [137].

The addition of 0.3% purple corn pigment containing pelargonidin, peonidin, cyanidin, malvidin, petunidin, and delphinidin to milk samples resulted in a retarding action on unsaturated fatty acids oxidation during a 7 day milk storage period. Anthocyanins could inhibit the oxidation of Unsaturated Fatty Acids (UFAs) by chain radical termination, acting as hydrogen atom donors to the peroxy radical; specifically, pelargonidin and petunidin significantly affected C14: 1*n*-5, C17: 1*n*-7, C18: 2*n*-6, C20: 2*n*-6, C20: 3*n*-3, and C20: 4*n*-6 degradation as well as cyanidin and total anthocyanins affected C14: 1*n*-5, C16: 1*n*-7, C17: 1*n*-7 [138].

Finally, recent studies were performed in order to obtain anthocyanin fractions from colored corns for the production of functional foods with higher resistant starch content. Blue corn extract, rich in cyanidin-3-O-(6″-O-malonylglucoside) was able to modify the relation of starch fractions of commercial maize starch contributing to a higher resistant starch formation, probably due to anthocyanins-starch interactions. Therefore, the amylolytic enzymatic hydrolysis in the small intestine could be limited and anthocyanins could be released in colon where the fermentation process led to molecules able to prevent colon cancer [139]. Similar results were also obtained testing flours from three different pigmented varieties when cooked. In fact, after in vitro starch digestion step higher resistant starch content was detected in colored samples in respect to a yellow variety, as well as lower starch hydrolysis index. Moreover, in this research, anthocyanins have been indicated as compounds able to modulate the enzyme activity and starch digestibility [140].

## 7. Non-Food Uses

Pigmented corn can be used in dry-grind ethanol process both in conventional starch hydrolyzing enzymes and in granular starch hydrolyzing enzymes process without any negative effect of the fermentation, as reported by Wang et al. [141] in 2016. In fact, for pigmented varieties similar ethanol conversion efficiencies to those of yellow corn were registered (75.1 ± 0.2% for yellow dent corn, 74.3 ± 0.4% for red corn, 78.4 ± 0.5% for blue corn, and 81.2 ± 1.0% for purple corn). Different results were obtained by Somavat et al. [20]: In fact, 14.3% (*v*/*v*) vs. 17.2% (*v*/*v*) final ethanol concentration were obtained for blue corn and yellow dent corn, respectively, in dry-grind process. Wet-milling and dry-grind characteristics of pigmented corn were also considered. In wet-milling, lower starch yields were registered for purple and blue corn than for yellow dent corn and in dry-milling, blue corn gave the lowest total endosperm yield. Anyway, purple and blue corn can potentially be used in all the three milling processes.

Ethanol production was estimated 42, 37, and 35.2 million gallon/yr for yellow, blue, and purple corn, respectively, as reported by Somavat et al. [142] after a commercial scale economic and technical feasibility analysis of dry grind process using pigmented corn to produce simultaneously ethanol and anthocyanins.

Purple corn extract was also used to develop an antimicrobial food packaging film. It was incorporated together with silver nanoparticles (AgNPs) into chitosan to develop active and intelligent food packaging films through hydrogen bonds (differently from silver nanoparticles which linked to chitosan by a coordination effect). Chitosan/AgNPs/purple corn extract film exhibited a higher barrier than chitosan/AgNPs or chitosan/purple corn extract. High antioxidant and antimicrobial properties were also registered due to synergistic effect between corn extract and AgNPs. The only disadvantage could be the changing color of films depending on pH value because of high level in anthocyanins present in purple corn extract (PCE). Anyway, chitosan/AgNPs/PCE film could be considered a novel active and intelligent food packaging material in food industry [143]. 

Another non-food interesting use of pigmented corn is the use of the anthocyanin fraction from husk, cob and silk of purple corn as photosensitizers in dye sensitized solar cells. The highest efficiency was obtained from purple corn husk extracted by acetone. The main characteristics of these solar cells are the high performance, the low cost, and the fact that they are environmentally friendly [144].

## 8. Conclusions

In the last years, the use of pigmented corns and the recycle of their waste represented an important issue for human health. In fact, many secondary metabolites belonging to different chemical classes of compounds have been detected in colored corn (seed, kernel, corn, husk, and bran derived-products) using chromatographic techniques and also many innovative approaches for the extraction of these molecules have been set up. The stability of the extracts deriving from colored corns is a key point in their potential use as food-derived products and this review also highlighted the different approached used to stabilize them. Finally, the main bioactivities registered in vitro and in vivo in animal models have been discussed.

## Figures and Tables

**Table 1:** Secondary metabolites of colored corn (kernel, cob, and flour and derived products). If not specified, the extraction method consisted in a solid/liquid extraction method.

Compound	Sample	Extraction Solvent	Reference
Cyanidin	Purple corn pericarp	H_2_O, Accelerated Solvent Extraction	[77]
Cyanidin-3-*O*-glucoside	Purple corn cob	Acidified (0.01% HCl 6N) 70% acetone	[11,20]
		Acidified (1% citric acid) MeOH	[21]
		MeOH	[30]
		Three-sequential step: Supercritical CO_2_, EtOH, water	[31]
		Acidified (0.01% HCl 6N) 70% acetone or MeOH or H_2_O or 20% EtOH or 40% EtOH or 50% EtOH or 60% EtOH or 80% EtOH or EtOH	[51]
		60–100% EtOH, *w*/*w*, ultrasound assisted extraction	[62]
		H_2_O or EtOH or H_2_O:EtOH, 1:1, *v*/*v*, ultrasonic assisted extraction	[63]
		MeOH followed by EtOH	[68]
		Acidified (0.01% HCl 6N) 40% EtOH or H_2_O or 70% acetone	[72]
		30% EtOH	[76]
		Aqueous EtOH	[78]
	Purple corn cob and husk	80% EtOH (*v*/*v*), maceration 80% EtOH (*v*/*v*), ultra-sound assisted extraction 80% EtOH (*v*/*v*), microwave-assisted extraction	[55]
	Purple corn kernel	EtOH-H_2_O, 30:70, *v*/*v*	[79]
		35% EtOH	[61]
	Purple corn pericarp	H_2_O or H_2_O + 0.2% SO_2_ or H_2_O + 0.5% lactic acid or H_2_O + 0.2% SO_2_ + 0.5% lactic acid	[58]
		H_2_O, Accelerated Solvent Extraction	[70,71,77,80,81,82]
	Purple waxy corn kernel	Acidified (0.01% HCl) 70% acetone	[83]
	Purple corn seeds	Acidified (1% TFA) MeOH	[34]
	Purple corn bran	Acidified (0.01% citric acid) 95% EtOH, ultrasound-assisted extraction	[53]
	Purple corn husk	0.1M HCl	[25]
	Raw and germinated purple corn	Acidified (1% TFA) 80% MeOH	[22]
	Red purple corn	Acidified (2% formic acid) water	[37]
	Purple, red corn kernel	2% aqueous formic acid	[84]
	Purple, red, blue corn pericarp	70% aqueous acetone	[85]
		Acidified (0.1M HCl) 80% MeOH	[50]
	Purple, red, blue corn flour	MeOH acidified 1% HCl	[18]
	Dark red, dark blue, light blue kernel flour	Boiling in 2N HCl 40 min, addition isoamyl alcohol, drying, suspension in MeOH	[44]
	Blue corn bean	Acidified (0.1% HCl) EtOH	[86]
	Blue corn tortillas	Acidified (citric acid 1M) EtOH	[87]
	Blue kernel flour	Acidified MeOH	[42]
	Blue corn flour, dough, polvorones	Acidified (1% TFA) MeOH	[88]
	Blue kernels and their tortillas	Acidified (0.05% HCl) MeOH	[33]
Cyanidin-3-*O*-(6″-*O*-malonylglucoside)	Purple corn cob	Acidified (0.01% HCl 6N) 70% acetone	[11,20]
		Acidified (0.01% HCl 6N) 70% acetone or MeOH or H_2_O or 20%EtOH or 40% EtOH or 50% EtOH or 60% EtOH or 80% EtOH or EtOH	[51]
		60–100% EtOH, *w*/*w*, ultrasound assisted extraction	[62]
		H_2_O or EtOH or H_2_O:EtOH, 1:1, *v*/*v*, ultrasonic assisted extraction	[63]
		MeOH followed by EtOH	[68]
		Acidified (0.01% HCl 6N) 40% EtOH or H_2_O or 70% acetone	[72]
	Purple corn kernel	EtOH-H_2_O, 30:70, *v*/*v*	[79]
		35% EtOH	[61]
	Purple corn pericarp	H_2_O or H_2_O + 0.2% SO_2_ or H_2_O + 0.5% lactic acid or H_2_O + 0.2% SO_2_ + 0.5% lactic acid	[58]
		H_2_O, Accelerated Solvent Extraction	[70,71,77,81,82]
	Purple corn seeds	Acidified (1% TFA) MeOH	[34]
	Purple corn bran	Acidified (0.01% citric acid) 95% EtOH, ultrasound-assisted extraction	[53]
	Purple corn husk	0.1M HCl	[25]
	Raw and germinated purple corn	Acidified (1% TFA) 80% MeOH	[22]
	Red purple corn	Acidified (2% formic acid) water	[37]
	Purple, red corn kernel	2% aqueous formic acid	[84]
	Purple, red, blue corn flour	MeOH acidified 1% HCl	[18]
	Purple, red, blue corn pericarp	Acidified (0.1M HCl) 80% MeOH	[50]
	Red, blue corn pericarp	70% aqueous acetone	[85]
	Blue corn bean	Acidified (0.1% HCl) EtOH	[86]
	Blue corn tortillas	Acidified (citric acid 1M) EtOH	[87]
	Blue kernels and their tortillas	Acidified (0.05% HCl) MeOH	[33]
	Blue corn flour, dough, polvorones	Acidified (1% TFA) MeOH	[88]
	Corn cob and husk	80% EtOH (*v*/*v*), maceration 80% EtOH (*v*/*v*), ultra-sound assisted extraction 80% EtOH (*v*/*v*), microwave-assisted extraction	[55]
Cyanidin-3-*O*-(3″-*O*-malonylglucoside)	Purple corn cob	Acidified (0.01% HCl) 70% acetone	[20]
	Purple corn flour	MeOH acidified 1% HCl	[18]
	Purple corn husk	0.1M HCl	[25]
	Blue kernels and their tortillas	Acidified (0.05% HCl) MeOH	[33]
Cyandin-3-*O*-(6″-*O*-ethylmalonylglucoside)	Purple corn cob	H_2_O or EtOH or H_2_O:EtOH, 1:1, *v*/*v*, ultrasonic assisted extraction	[63]
	Blue corn tortillas	Acidified (citric acid 1M) EtOH	[87]
Cyanidin-3-*O*-(6″-acetylgalactoside)	Blue corn tortillas	Acidified (citric acid 1M) EtOH	[87]
Cyanidin-3-*O*-(6″-piruvoylglucoside)	Blue corn tortillas	Acidified (citric acid 1M) EtOH	[87]
Cyanidin-3-*O*-glucoside monomalonate	Purple corn husk	0.1M HCl	[25]
Cyanidin-3-*O*-malonylhexoside	Raw and germinated purple corn	Acidified (1% TFA) 80% MeOH	[22]
	Blue kernels and their tortillas	Acidified (0.05% HCl) MeOH	[33]
Cyanidin-3-*O*-(3″-6″-dimalonylglucoside)	Purple corn cob	Acidified (0.01% HCl) 70% acetone	[20]
	Purple corn pericarp	H_2_O, Accelerated Solvent Extraction	[77]
	Purple corn husk	0.1M HCl	[25]
	Raw and germinated purple corn	Acidified (1% TFA) 80% MeOH	[22]
	Purple, red, blue corn pericarp	Acidified (0.1M HCl) 80% MeOH	[50]
	Blue corn flour, dough, polvorones	Acidified (1% TFA) MeOH	[88]
Cyanidin-3-*O*-glucoside-dimalonate	Purple corn husk	0.1M HCl	[25]
Cyanidin-3-*O*-(6″-succinylglucoside)	Purple corn cob	Acidified (0.01% HCl 6N) 70% acetone	[11]
	Blue corn bean	Acidified (0.1% HCl) EtOH	[86]
	Blue kernels and their tortillas	Acidified (0.05% HCl) MeOH	[33]
	Blue kernel flour	Acidified MeOH	[42]
Cyanidin-3-*O*-(6″-caffeoilglucoside)	Blue corn bean	Acidified (0.1% HCl) EtOH	[86]
Cyanidin-3-*O*-(6″-disuccinylglucoside)	Blue kernel flour	Acidified MeOH	[42]
	Blue kernels and their tortillas	Acidified (0.05% HCl) MeOH	[33]
Cyanidin-3,5-diglucoside	Raw and germinated purple corn	Acidified (1% TFA) 80% MeOH	[22]
	Red purple corn	Acidified (2% formic acid) water	[37]
	Blue corn tortillas	Acidified (citric acid 1M) EtOH	[87]
Pelargonidin-3-*O*-glucoside	Purple corn cob	Acidified (0.01% HCl 6N) 70% acetone	[11,20]
		Acidified (1% citric acid) MeOH	[21]
		MeOH	[30]
		Three-sequential step: Supercritical CO_2_, EtOH, water	[31]
		Acidified (0.01% HCl 6N) 70% acetone or MeOH or H_2_O or 20%EtOH or 40% EtOH or 50% EtOH or 60% EtOH or 80% EtOH or EtOH	[51]
		H_2_O or EtOH or H_2_O:EtOH, 1:1, *v*/*v*, ultrasonic assisted extraction	[63]
		MeOH followed by EtOH	[68]
		Acidified (0.01% HCl 6N) 40% EtOH or H_2_O or 70% acetone	[72]
		30% EtOH	[76]
		Aqueous EtOH	[78]
		60–100% EtOH, *w*/*w*, ultrasound assisted extraction	[62]
		MeOH followed by EtOH	[68]
	Purple corn cob and husk	80% EtOH (*v*/*v*), maceration 80% EtOH (*v*/*v*), ultra-sound assisted extraction 80% EtOH (*v*/*v*), microwave-assisted extraction	[55]
	Purple corn kernel	EtOH-H_2_O, 30:70, *v*/*v*	[79]
		35% EtOH	[61]
	Purple corn pericarp	H_2_O or H_2_O + 0.2% SO_2_ or H_2_O + 0.5% lactic acid or H_2_O + 0.2% SO_2_ + 0.5% lactic acid	[58]
		H_2_O, Accelerated Solvent Extraction	[70,71,77,80,81,82]
	Purple corn seeds	Acidified (1% TFA) MeOH	[34]
	Purple corn bran	Acidified (0.01% citric acid) 95% EtOH, ultrasound-assisted extraction	[53]
	Purple corn husk	0.1M HCl	[25]
	Purple corn flour	MeOH acidified 1% HCl	[18]
	Raw and germinated purple corn	Acidified (1% TFA) 80% MeOH	[22]
	Purple, red corn pericarp	70% aqueous acetone	[85]
	Purple, red corn kernel	2% aqueous formic acid	[84]
	Purple, red, blue corn pericarp	Acidified (0.1M HCl) 80% MeOH	[50]
	Purple waxy corn kernel	Acidified (0.01% HCl) 70% acetone	[83]
	Dark red, dark blue, light blue kernel flour	Boiling in HCl 2N 40 min, addition isoamyl alcohol, drying, suspension in MeOH	[44]
	Blue corn bean	Acidified (0.1% HCl) EtOH	[86]
	Blue kernel flour	Acidified MeOH	[42]
	Blue kernels and their tortillas	Acidified (0.05% HCl) MeOH	[33]
	Blue corn flour, dough, polvorones	Acidified (1% TFA) MeOH	[88]
			
Pelargonidin-3-*O*-(6″-malonylglucoside)	Purple corn cob	Acidified (0.01% HCl 6N) 70% acetone	[11]
		Acidified (0.01% HCl) 70% acetone	[20]
		H_2_O or EtOH or H_2_O:EtOH, 1:1, *v*/*v*, ultrasonic assisted extraction	[63]
	Purple corn flour	MeOH acidified 1% HCl	[18]
	Raw and germinated purple corn	Acidified (1% TFA) 80% MeOH	[22]
	Blue kernels and their tortillas		
	Purple corn seeds	Acidified (1% TFA) MeOH	[34]
	Red purple corn	2% aqueous formic acid	[37]
	Purple corn cob	Acidified (0.01% HCl 6N) 70% acetone or MeOH or H_2_O or 20% EtOH or 40% EtOH or 50% EtOH or 60% EtOH or 80% EtOH or EtOH	[51]
	Purple corn bran	Acidified (0.01% citric acid) 95% EtOH, ultrasound-assisted extraction	[53]
	Purple corn husk	0.1M HCl	[25]
	Purple corn pericarp	H_2_O or H_2_O + 0.2% SO_2_ or H_2_O + 0.5% lactic acid or H_2_O + 0.2% SO_2_ + 0.5% lactic acid	[58]
	Purple corn kernel	35% EtOH	[61]
	Purple corn	60–100% EtOH, *w*/*w*, ultrasound assisted extraction	[62]
	Purple corn cob and husk	80% EtOH (*v*/*v*), maceration 80% EtOH (*v*/*v*), ultra-sound assisted extraction 80% EtOH (*v*/*v*), microwave-assisted extraction	[55]
	Purple corn pericarp	H_2_O, Accelerated Solvent Extraction	[70,71,77,81,82]
	Purple corn cob	Acidified (0.01% HCl 6N) 40% EtOH or H_2_O or 70% acetone	[72]
	Blue corn bean	Acidified (0.1% HCl) EtOH	[86]
	Purple, red, blue corn pericarp	70% aqueous acetone	[85]
		Acidified (0.1M HCl) 80% MeOH	[50]
	Purple, red corn kernel	2% aqueous formic acid	[84]
	Blue corn flour, dough, polvorones	Acidified (1% TFA) MeOH	[88]
			
Pelargonidin-3-*O*-(3″-malonylglucoside)	Purple corn husk	0.1M HCl	[25]
Pelargonidin-3-*O*-(3″-6″-dimalonylglucoside)	Purple corn seeds	Acidified (1% TFA) MeOH	[34]
	Purple corn husk	0.1M HCl	[25]
	Purple, red, blue corn pericarp	Acidified (0.1M HCl) 80% MeOH	[50]
	Raw and germinated purple corn	Acidified (1% TFA) 80% MeOH	[22]
Pelargonidin-3,5-diglucoside	Red purple corn	2% aqueous formic acid	[37]
Peonidin-3-*O*- glucoside	Purple corn cob	Acidified (0.01% HCl 6N) 70% acetone	[11,20]
		Acidified (1% citric acid) MeOH	[21]
		MeOH	[30]
		Three-sequential step: Supercritical CO_2_, EtOH, water	[31]
		Acidified (0.01% HCl 6N) 70% acetone or MeOH or H_2_O or 20% EtOH or 40% EtOH or 50% EtOH or 60% EtOH or 80% EtOH or EtOH	[51]
		60–100% EtOH, *w*/*w*, ultrasound assisted extraction	[62]
		H_2_O or EtOH or H_2_O:EtOH, 1:1, *v*/*v*, ultrasonic assisted extraction	[63]
		MeOH followed by EtOH	[68]
		Acidified (0.01% HCl 6N) 40% EtOH or H_2_O or 70% acetone	[72]
		30% EtOH	[76]
		Aqueous EtOH	[78]
	Purple corn kernel	EtOH-H_2_O, 30:70, *v*/*v*	[79]
		35% EtOH	[61]
	Purple corn pericarp	H_2_O or H_2_O + 0.2% SO_2_ or H_2_O + 0.5% lactic acid or H_2_O + 0.2% SO_2_ + 0.5% lactic acid	[58]
		H_2_O, Accelerated Solvent Extraction	[70,71,77,80,81,82]
	Purple corn seeds	Acidified (1% TFA) MeOH	[34]
	Purple corn bran	Acidified (0.01% citric acid) 95% EtOH, ultrasound-assisted extraction	[53]
	Purple corn husk	0.1M HCl	[25]
	Purple corn flour	MeOH acidified 1% HCl	[18]
	Raw and germinated purple corn	Acidified (1% TFA) 80% MeOH	[22]
	Purple waxy corn kernel	Acidified (0.01% HCl) 70% acetone	[83]
	Red purple corn	2% aqueous formic acid	[37]
	Purple, red corn kernel	2% aqueous formic acid	[84]
	Purple, red, blue corn pericarp	70% aqueous acetone	[89]
		Acidified (0.1M HCl) 80% MeOH	[50]
	Dark red, dark blue, light blue kernel flour	Boiling in HCl 2N, addition isoamyl alcohol, drying, suspension in MeOH	[44]
	Blue kernel flour	Acidified MeOH	[42]
	Corn cob and husk	80% EtOH (*v*/*v*), maceration 80% EtOH (*v*/*v*), ultra-sound assisted extraction 80% EtOH (*v*/*v*), microwave-assisted extraction	[55]
Peonidin-3-*O*-(6″-malonylglucoside)	Purple corn cob	Acidified (0.01% HCl 6N) 70% acetone	[11,20]
		Acidified (0.01% HCl 6N) 70% acetone or MeOH or H_2_O or 20% EtOH or 40% EtOH or 50% EtOH or 60% EtOH or 80% EtOH or EtOH	[51]
		60–100% EtOH, *w*/*w*, ultrasound assisted extraction	[62]
		H_2_O or EtOH or H_2_O:EtOH, 1:1, *v*/*v*, ultrasonic assisted extraction	[63]
		MeOH followed by EtOH	[68]
		Acidified (0.01% HCl 6N) 40% EtOH or H_2_O or 70% acetone	[72]
	Purple corn kernel	EtOH-H_2_O, 30:70, *v*/*v*	[79]
	Purple corn pericarp	H_2_O or H_2_O + 0.2% SO_2_ or H_2_O + 0.5% lactic acid or H_2_O + 0.2% SO_2_ + 0.5% lactic acid	[58]
		H_2_O, Accelerated Solvent Extraction	[70,71,77,81,82]
	Purple corn seeds	Acidified (1% TFA) MeOH	[34]
	Purple corn husk	0.1M HCl	[25]
	Purple corn flour	MeOH acidified 1% HCl	[18]
	Raw and germinated purple corn	Acidified (1% TFA) 80% MeOH	[22]
	Purple, red, blue corn pericarp	70% aqueous acetone	[85]
		Acidified (0.1M HCl) 80% MeOH	[50]
	Corn cob and husk	80% EtOH (*v*/*v*), maceration 80% EtOH (*v*/*v*), ultra-sound assisted extraction 80% EtOH (*v*/*v*), microwave-assisted extraction	[55]
Peonidin-3-*O*-(3″-6″-dimalonylglucoside)	Purple corn cob	Acidified (0.01% HCl 6N) 70% acetone	[11]
	Purple corn husk	0.1M HCl	[25]
	Red purple corn	2% aqueous formic acid	[37]
	Purple, red corn kernel	2% aqueous formic acid	[84]
	Purple, red, blue corn pericarp	Acidified (0.1M HCl) 80% MeOH	[50]
Peonidin-3,5-diglucoside	Red purple corn	2% aqueous formic acid	[37]
Petunidin-3-*O*-glucoside	Purple corn cob	H_2_O or EtOH or H_2_O:EtOH, 1:1, *v*/*v*, ultrasonic assisted extraction	[63]
Malvidin-3-*O*-(6″-dimalonylglucoside)	Purple corn	60–100% EtOH, *w*/*w*, ultrasound assisted extraction	[62]
Malvidin-3-*O*-(6″-malon-*p*-hydroxybenzoilglucoside)	Purple corn	60–100% EtOH, *w*/*w*, ultrasound assisted extraction	[62]
Delphinidin-3-*O*-(6″-malonylglucoside)	Raw and germinated purple corn	Acidified (1% TFA) 80% MeOH	[22]
Delphinidin-3-*O*-(6″-*O*-*p*-coumaroylglucoside)	Purple corn pericarp	H_2_O, Accelerated Solvent Extraction	[77]
Catechin-(4,8)-cyanidin-3-glucoside	Purple corn seeds	Acidified (1% TFA) MeOH	[34]
Catechin-(4,8)-cyanidin-3,5-diglucoside	Purple corn cob	Acidified (0.01% HCl 6N) 70% acetone	[11]
	Purple corn pericarp	H_2_O, Accelerated Solvent Extraction	[77,81]
	Purple corn seeds	Acidified (1% TFA) MeOH	[34]
	Raw and germinated purple corn	Acidified (1% TFA) 80% MeOH	[22]
	Red purple corn	2% aqueous formic acid	[37]
Catechin-(4,8)-cyanidin-3-malonylglucoside	Purple corn seeds	Acidified (1% TFA) MeOH	[34]
Epicatechin-(4,8)-cyanidin-3-malonylglucoside	Purple corn seeds	Acidified (1% TFA) MeOH	[34]
(Epi)catechin (4-8)-cyanidin 3-malonylglucoside-5 glucoside	Purple corn seeds	Acidified (1% TFA) MeOH	[34]
	Raw and germinated purple corn	Acidified (1% TFA) 80% MeOH	[22]
	Red purple corn	2% aqueous formic acid	[37]
Afzelechin-(4-8)-cyanidin 3,5 diglucoside	Red purple corn	2% aqueous formic acid	[37]
Catechin-(4,8)-pelargonidin-3-glucoside	Purple corn seeds	Acidified (1% TFA) MeOH	[34]
(Epi)catechin (4-8)-pelargonidin 3,5 diglucoside	Raw and germinated purple corn	Acidified (1% TFA) 80% MeOH	[22]
	Red purple corn	2% aqueous formic acid	[37]
Catechin (4-8)-pelargonidin 3-malonylglucoside-5 glucoside	Red purple corn	2% aqueous formic acid	[37]
Afzelechin-(4-8)-pelargonidin 3,5 diglucoside	Red purple corn	2% aqueous formic acid	[37]
Afzelechin- (4-8)-pelargonidin 3-malonylglucoside-5 glucoside	Red purple corn	2% aqueous formic acid	[37]
Catechin-(4,8)-peonidin-3-glucoside	Purple corn seeds	Acidified (1% TFA) MeOH	[34]
Epicatechin-(4,8)-peonidin-3-glucoside	Purple corn seeds	Acidified (1% TFA) MeOH	[34]
Catechin (4-8)-peonidin- 3,5 diglucoside	Red purple corn	2% aqueous formic acid	[37]
Catechin (4-8)-peonidin 3-malonylglucoside-5 glucoside	Red purple corn	2% aqueous formic acid	[37]
Afzelechin-(4-8)-peonidin-3,5 diglucoside	Red purple corn	2% aqueous formic acid	[37]
Proanthocyanidin dimer	Blue corn tortillas	Acidified (citric acid 1M) EtOH	[87]
Proanthocyanidin trimer	Blue corn tortillas	Acidified (citric acid 1M) EtOH	[87]
Proanthocyanidin tetramer	Blue corn tortillas	Acidified (citric acid 1M) EtOH	[87]
*p*-hydroxycinnamic acid	Purple corn kernel	EtOH-H_2_O, 30:70, *v*/*v*	[79]
		35% EtOH	[61]
Caffeic acid	Purple corn cob	30% EtOH	[76]
	Purple corn pericarp	H_2_O, Accelerated Solvent Extraction	[82]
	Purple waxy corn kernel	80% EtOH	[83]
	Purple, red, blue corn flour	Acidified (1% HCl) MeOH	[18]
	Blue corn bean	Acidified (0.1% HCl) EtOH	[86]
Caffeic acid derivatives	Purple corn	Acidified (0.1% HCl) MeOH:acetone:H_2_O, 45:45:10, *v*/*v*/*v*	[90]
Caffeic acid 4-*O*-hexoside	Red corn cob	55% EtOH, ultrasonic-assisted extraction	[54]
Ferulic acid	Purple corn cob	Acidified (0.1% HCl) MeOH:acetone:H_2_O, 45:45:10, *v*/*v*/*v* + NaOH 3N + ethyl acetate	[90]
		MeOH	[30]
		30% EtOH	[76]
	Purple corn kernel	EtOH-H_2_O, 30:70, *v*/*v*	[79]
		35% EtOH	[61]
	Purple corn pericarp	H_2_O, Accelerated Solvent Extraction	[82]
	Purple waxy corn kernel	80% EtOH	[83]
	Purple corn flour, orange kernel	80% MeOH	[69,91]
	Purple, red, blue corn flour	Acidified (1% HCl) MeOH	[18]
Ferulic acid derivatives	Purple corn cob	Acidified (0.1% HCl) MeOH:acetone:H2O, 45:45:10, *v*/*v*/*v* + NaOH 3N + ethyl acetate	[90]
		Aqueous EtOH	[78]
3-*O*-feruloylquinic acid	Raw purple corn	Acidified (1% TFA) 80% MeOH	[22]
5-*O*-feruloylquinic acid	Purple corn cob	Aqueous EtOH	[78]
Feruloyl-caffeoylglycerol	Purple, red, blue corn flour	Acidified (1% HCl) MeOH	[18]
N,N′-diferuloylputrescine	Purple, red, blue corn flour	Acidified (1% HCl) MeOH	[18]
*o*-coumaric acid	Purple corn pericarp	H_2_O, Accelerated Solvent Extraction	[26]
*p*-coumaric acid	Orange kernel	80% MeOH	[69]
	Purple corn cob	Acidified (0.1% HCl) MeOH:acetone:H_2_O, 45:45:10, *v*/*v*/*v* + NaOH 3N + ethyl acetate	[90]
	Purple corn cob	30% EtOH	[76]
	Purple waxy corn kernel	80% EtOH	[83]
	Red corn cob	55% EtOH, ultrasonic-assisted extraction	[54]
	Blue corn bean	Acidified (0.1% HCl) EtOH	[86]
*p*-coumaroylquinic acid	Green, pinky, purple corn silk	95% MeOH	[27]
*p*-coumaric acid derivatives	Purple corn	Acidified (0.1% HCl) MeOH:acetone:H_2_O, 45:45:10, *v*/*v*/*v*	[90]
Coumaroyl-caffeoylglycerol	Purple, red, blue corn flour	Acidified (1% HCl) MeOH	[18]
N-coumaroyl-N′-feruloylputrescine	Purple, red, blue corn flour	Acidified (1% HCl) MeOH	[18]
N,N′-dicoumaroylspermidine	Purple, red, blue corn flour	Acidified (1% HCl) MeOH	[18]
Protocatechuic acid	Purple corn cob	30% EtOH	[76]
	Purple corn flour	80% MeOH	[91]
	Purple corn kernel	EtOH-H_2_O, 30:70, *v*/*v*	[79]
		35% EtOH	[61]
	Purple corn pericarp	H_2_O, Accelerated Solvent Extraction	[82]
	Purple waxy corn kernel	80% EtOH	[83]
Sinapic acid	Purple corn cob	30% EtOH	[76]
Syringic acid	Purple corn cob	30% EtOH	[76]
		MeOH	[30]
3-*O*-caffeoylquinic acid	Raw and germinated purple corn	Acidified (1% TFA) 80% MeOH	[22]
	Green, pinky, purple corn silk	95% MeOH	[29]
4-*O*-caffeoylquinic acid	Green, pinky, purple corn silk	95% MeOH	[29]
5-*O*-caffeoylquinic acid	Green, pinky, purple corn silk	95% MeOH	[29]
	Red corn cob	55% EtOH, ultrasonic-assisted extraction	[54]
	Blue corn bean	Acidified (0.1% HCl) EtOH	[86]
Dicaffeoyl-succinoylquinic acid	Raw and germinated purple corn	Acidified (1% TFA) 80% MeOH	[22]
*p*-Hydroxybenzoic acid	Orange kernel	80% MeOH	[32]
	Raw purple corn	Acidified (1% TFA) 80% MeOH	[22]
	Purple corn cob	MeOH	[30]
		30% EtOH	[76]
	Purple waxy corn kernel	80% EtOH	[83]
Vanillic acid	Purple corn cob	MeOH	[30]
		30% EtOH	[76]
	Purple corn kernel	EtOH-H_2_O, 30:70, *v*/*v*	[79]
		35% EtOH	[61]
	Purple corn pericarp	H_2_O, Accelerated Solvent Extraction	[82]
	Raw and germinated purple corn	Acidified (1% TFA) 80% MeOH	[22]
	Purple waxy corn kernel	80% EtOH	[83]
Vanillic acid hexoside	Germinated purple corn	Acidified (1% TFA) 80% MeOH	[22]
2,4,6-tribenzoic acid	Purple corn kernel	EtOH-H_2_O, 30:70, *v*/*v*	[79]
		35% EtOH	[61]
Kaempferol	Purple corn pericarp	H_2_O, Accelerated Solvent Extraction	[82]
Kaempferol *O*-hexoside	Germinated purple corn	Acidified (1% TFA) 80% MeOH	[22]
Kaempferol-3-*O*-glucoside	Raw purple corn	Acidified (1% TFA) 80% MeOH	[22]
Kaempferol-7-*O*-glucoside	Purple corn cob	Aqueous EtOH	[78]
Kaempferol acetylhexoside	Raw and germinated purple corn	Acidified (1% TFA) 80% MeOH	[22]
Keampfero-3,7-di-*O*-hexoside	Purple corn cob	Aqueous EtOH	[78]
Kaempferol rhamnoside hexoside	Raw and germinated purple corn	Acidified (1% TFA) 80% MeOH	[22]
Keampferolo-7-*O*-(6″-malonylglucoside)	Purple corn cob	Aqueous EtOH	[78]
Kaempferol-7-*O*-rutinoside	Purple corn cob	Aqueous EtOH	[78]
kaempferol-3-*O*-hexosyl-7-*O*-glucuronilhexoside	Purple corn cob	Aqueous EtOH	[78]
Quercetin	Purple corn cob	30% EtOH	[76]
	Purple corn pericarp	H_2_O, Accelerated Solvent Extraction	[82]
Quercetin-*O*-glucoside	Raw and germinated purple corn	Acidified (1% TFA) 80% MeOH	[22]
Quecertin-7-*O*-glucoside	Purple corn cob	Aqueous EtOH	[78]
Quercetin 3-*O*-hexoside	Raw and germinated purple corn	Acidified (1% TFA) 80% MeOH	[22]
Quercetin-3-*O*-rutinoside	Purple corn cob	30% EtOH	[76]
	Purple corn pericarp	H_2_O, Accelerated Solvent Extraction	[77,82]
	Raw and germinated purple corn	Acidified (1% TFA) 80% MeOH	[22]
Quecertin-7-*O-p*-coumaroylhexoside	Purple corn cob	Aqueous EtOH	[78]
Myricetin-7-*O*-hexoside	Purple corn cob	Aqueous EtOH	[78]
Myricetin-3,7-di-*O*-glucoside	Purple corn cob	Aqueous EtOH	[78]
Isorhamnetin-*O*-hexoside	Raw purple corn	Acidified (1% TFA) 80% MeOH	[22]
	Purple corn cob	Aqueous EtOH	[78]
Isorhamnetin-3-*O*-glucoside	Raw and germinated purple corn	Acidified (1% TFA) 80% MeOH	[22]
Isorhamnetin-3-*O*-rutinoside	Raw and germinated purple corn	Acidified (1% TFA) 80% MeOH	[22]
Isorhamnetin-7-*O*-rutinoside	Purple corn cob	Aqueous EtOH	[78]
Isorhamnetin-3,7-di-*O*-hexiside	Purple corn cob	Aqueous EtOH	[78]
Isorhamentin-3-*O*-hexosyl-7-*O*-glucuronilhexoside	Purple corn cob	Aqueous EtOH	[78]
Apigenin-*O*-hexoside	Red corn cob	55% EtOH, ultrasonic-assisted extraction	[54]
	Blue corn bean	Acidified (0.1% HCl) EtOH	[86]
Apigenin-7-*O*-glucoside	Purple corn cob	Aqueous EtOH	[78]
Apigenin-O-pentosyl hexoside	Red corn cob	55% EtOH, ultrasonic-assisted extraction	[54]
di-C,C-hexosyl-apigenin	Red purple corn	2% aqueous formic acid	[37]
C-hexosyl apigenin	Red purple corn	2% aqueous formic acid	[37]
C-hexosyl-C-pentosyl apigenin	Red purple corn	2% aqueous formic acid	[37]
	Red corn cob	55% EtOH, ultrasonic-assisted extraction	[54]
Luteolin	Purple corn pericarp	H_2_O, Accelerated Solvent Extraction	[82]
Luteolin-7-*O*-glucoside	Purple corn cob	Aqueous EtOH	[78]
Luteolin-*O*-rutinoside	Red corn cob	55% EtOH, ultrasonic-assisted extraction	[54]
2″-*O*-pentoside-8-C-hexoside Luteolin	Raw and germinated purple corn	Acidified (1% TFA) 80% MeOH	[22]
Naringenin	Purple corn pericarp	H_2_O, Accelerated Solvent Extraction	[77,82]
Daidzin	Red corn cob	55% EtOH, ultrasonic-assisted extraction	[54]
	Blue corn bean	Acidified (0.1% HCl) EtOH	[86]
Procyanidin dimer	Red corn cob	55% EtOH, ultrasonic-assisted extraction	[54]
Scopoletin	Red corn cob	55% EtOH, ultrasonic-assisted extraction	[54]
Maysin	Green, pinky, purple corn silk	95% MeOH	[25]
Maysin derivative	Green, pinky, purple corn silk	95% MeOH	[25]
Methoxymaysin derivative	Green, pinky, purple corn silk	95% MeOH	[25]
Vitexin	Red purple corn	2% aqueous formic acid	[37]
Isovitexin	Red purple corn	2% aqueous formic acid	[37]
Hirsutrin	Purple corn kernel	EtOH-H_2_O, 30:70, *v*/*v*	[79]
		35% EtOH	[61]
3′-methoxyhirsutrin	Purple corn kernel	EtOH-H_2_O, 30:70, *v*/*v*	[79]
		35% EtOH	[61]
GABA	Germinated purple corn	H_2_O + derivatization by phenyl isothiocyanate	[22]
Lutein	Orange kernel	H_2_O-saturated butan-1-ol	[69,92]
	Red kernel	Diethyl ether:petroleum ether, 2:1, *v*/*v*	[47]
Zeaxanthin	Orange kernel	H_2_O-saturated butan-1-ol	[69,92]
	Red kernel	Diethyl ether:petroleum ether, 2:1, *v*/*v*	[47]
β-Cryptoxanthin	Orange kernel	Water-saturated butan-1-ol	[69,92]
	Red kernel	Diethyl ether:petroleum ether, 2:1, *v*/*v*	[47]
β-Carotene	Orange kernel	H_2_O-saturated butan-1-ol	[69,92]
	Red kernel	Diethyl ether:petroleum ether, 2:1, *v*/*v*	[47]

**Table 2:** A summary of the in vitro and in vivo studies correlated the healthy properties discussed in Section 5.

Type of Trial	Metabolite, Origin	Extract/Dose/Period of Treatment	Main Biological Effect	Reference
In vitro anti-bacterial activity (*Lactobacillus helveticus*, *Bifidobacterium longum*, *Helicobacter pylori*)	Free and bound phenolic compounds, Peruvian purple corn	free phenolic fraction 10–50 mg/mL, bound phenolic fraction 10 mg/mL	No antibacterial activity	[79]
In vitro antibacterial activity against pathogenic bacteria (*Pseudomonas aeruginosa*, *Escherichia coli*, *Bacillus subtilis* and *Staphylococcus aureus*)	Polyphenolic extracts from corn germplasm, Chorotega and Brunca Regions of Costa Rica	100 mg/mL uncolored and colored extracts	Antibacterial activity of purple extract only against *B. subtilis*	[96]
In vitro antimutagenic activity (TA100 and TA98 *Salmonella typhimurium* strains)	Flavonoid and anthocyanin extracts, red and blue Mexican corn	Flavonoid extract: 7 and 138 mm (+)-catechin equivalents, anthocyanin extracts: 1.6 and 3.2 mm cyanidin-3-glucoside equivalents	No effect on bacterial strain; ↓ Aflatoxin B1 mutagenicity	[38,97]
In vitro inhibitory capacity of hyperglycemia and obesity target enzymes	Free phenolic fraction, twenty-two Peruvian corn samples corresponding to five corn races (Arequipeño, Cabanita, Kculli, Granada and Coruca)	50, 125, 250 mg dose for a-amylase assay; 5, 12.5, 25 mg dose for a -glucosidase assay; 2.5, 6.25, 12.5 mg dose for lipase assay	↑ α-glucosidase and a-amylase inhibitory activity; only Kculli race sample ↑ lipase inhibitory activity	[100]
In vitro inhibitory capacity of hyperglycemia and obesity target enzymes	Phytocomplex from Moradyn purple corn cob	0.062–1 mg/mL	↑ α-glucosidase inhibitory activity; no a-amylase inhibitory activity	[78]
In vitro response to adipogenesis, inflammation, and insulin resistance in 3T3-L1 adipocytes on basal and inflammatory conditions	Anthocyanin-rich extract from purple corn pericarp	0.4 mg/mL (50.3 μM C3G equivalent)	↓ Preadipocyte differentiation, fatty acid synthase enzymatic activity, PPAR-γ transcriptional activity on adipocytes in basal conditions TNF-α-dependent inflammatory status (↑ adiponectin, leptin), glucose uptake, and GLUT4 membrane translocation, ↓ reduced reactive oxygen species generation	[80]
In vitro cellular insulin secretion and hepatic glucose uptake (pancreatic cells and hepatocytes)	Anthocyanin-rich extract from purple corn pericarp	0.125-1 mg/mL	↑ Glucose-stimulated insulin secretion in INS-1E cells and activation of the free fatty acid receptor-1; ↑ glucose uptake in HepG2 cells and activation of glucokinase	[80]
In vitro anti-inflammatory, anti-adipogenic and anti-diabetic activity (mouse 3T3-L1 preadipocytes and RAW264.7 macrophages)	Anthocyanin-rich extract from twenty genotypes of Apache Red purple corn pericarp	0.05–1.0 mg/mL in α-amylase and dipeptidyl peptidase-4 inhibitory assays; 1 mg/mL in the other assays	↑ Antiadipogenic effect by inhibiting preadipocyte differentiation and reducing TG content in mature adipocyte; Inhibition of α-amylase activity (IC50: 109.5–172.7 µg/mL), and DPP-IV activity (IC50: 65.5–702.7 µg/mL); The presence of Cyanidin, perlagonidin glycosylate forms, their malonilate derivatives, quercetin, luteolin and vanillic acid in corn extracts has been positively correlated with anti-adipogenic potential	[82]
In vitro anti-diabetic activity	Commercial purple corn kernel extract	IC50 26.12 µg/mL for phenolic acid and flavonoids fraction IC50 58.20 µg/mL for anthocyanin fraction	↓ Tyrosine phosphatase 1β expression	[61]
In vitro anti-obesity activity (murine 3T3-L1 cell line)	Purple corn silk extract	250–1000 μg/mL	↓ Adipogenesis (inhibition on adipocyte differentiation and reduction of total lipid accumulation); ↑ Lipolysis and apoptosis inductive effect (increase of released glycerol content and of nucleic condensing and apoptotic bodies)	[26]
In vivo (C57BL/KsJ db/db mice) anti-diabetic effect	Anthocyanin-rich purple corn extract	10 or 50 mg/kg/day for 8 weeks	Protection of pancreatic β-cells, increase of insulin secretion and AMPK activation in the liver (↓ blood glucose and HbA1c levels, ↑ C-peptide and adiponectin levels)	[79]
In vivo (rats) attenuation of diet-induced metabolic syndrome	Purple corn kernel flour	50 mg/kg/die (≈ 8mg anthocyanins/kg/die) with or without high fat or high carbohydrate diet for 8 weeks, after 8 weeks of only high fat or high carbohydrate diets	↓ Visceral adiposity index, total body fat mass, systolic blood pressure, plasma triacylglycerols, total cholesterol, blood glucose, HbA1c, and glucagone; improved glucose tolerance, liver, and cardiovascular structure and function	[103]
In vivo (Wistar rats) attenuation of diet-induced metabolic syndrome	Blue corn extract	2 mL/die (9.97 mg total polyphenols of which 2.92 mg of anthocyanins) with or without high-sucrose diet or high-sucrose diet plus cholesterol, for 4 weeks after 12 weeks of high-sucrose diet	↑ Enhanced HDL-C; ↓ Systolic blood pressure, serum triglycerides, total cholesterol, epididymal adipose tissue weight	[104]
In vivo (Sprague Dawley rats) amelioration of lipid metabolism	Purple corn extract	401 mg of anthocyanins per food kg, with olive or chia oil for 78 days	↑ Anti-adipogenic effect mediated by the upregulation of liver PPAR-α and the inhibition of SREPB-1C following the combined intake of Purple corn extract and Chia oil	[105]
In silico study to evaluate anti-diabetic effect	Blue corn extract	Total anthocyanins: 582 mg/kg DW (C3G)	Interactions between anthocyanins and target proteins involved in type 2 diabetes: cyanidin 3-glucoside with 11β-HS, GFAT, PPARG; delphinidin 3-glucoside with 11β-HS, GFAT, PTP and RTKs; petunidin 3-glucoside with 11β-HS and PTP	[86]
In vivo (streptozotocin-diabetic rats) effect on oxidative stress related disordersin diabetic condition	*Zingiber officinale* rhizomes and purple *Zea mays* seeds extract (1:4, *v*/*v*)	100–300 mg/kg/die for 21 days	↓ Diabetic neurophaty; Improved sciatic function index, paw withdrawal threshold intensity, paw withdrawal latency, nerve conduction velocity); ↓ Oxidative stress status (MDA, SOD, GPx, CAT, AR); ↑ Axon density in the lesion nerve.	[106]
In vitro anti-inflammatory properties	Wet milling, dry milling, and dry grind purple and red corn pericarp	1 mg/mL red corn dry milling pericarp proanthocyanidin 0.1–1 mg/mL purple corn dry milling pericarp proanthocyanidin	↓ Cyclooxygenase-2; ↓ Inducible nitric oxide synthase activities	[85]
In vitro effect on inflammatory paracrineinterplay between macrophages and adipocytes	Dry-milling purple or red corn pericarp extract	0.25–1 mg/mL purple or red pericarp extract	↓ Reduction of pro-inflammatory mediators expressed by LPS-induced magrophages or adipocytes exposed to macrophage-conditioned medium; ↓ Inhibition of NF-κB and JNK pro-inflammatory pathways; ↓ Insulin resistance caused by adipocyte-macrophage interaction under a metaflammation state	[107]
In vivo (C57BL/6 mice) effect on oxidative stress and inflammatory status associated to obesity	Purple corn extract	200 mg/kg with a high-fat diet, 12 weeks	↓ serum and hepatic lipid peroxidation (TG, TC, LDL, MDA, SOD, TNFα, IL-6, iNOS, NF-κB, GPx); ↑ fecal butyric acid levels	[89]
In vivo (rats) effect on liver oxidative stress	Raw and nixtamal blue corn	18 g/day, with and without high fat diet, 8 weeks	↓ Oxidative stress (MDA, ROS, SOD, GPx, CAT)	[108]
In vivo (sheep) effect on oxidative stress	Sunred NO.5 F purple corn pigment	Diet: 78% pigment, 30% EtOH, 2% citric acid, 14 days	↓ Serum lipid peroxidation (SOD, total antioxidant capacity, GSH, 8-HOdG); ↓ Oxidation resistance of plasma	[109]
In vivo (cows) effect on oxidative stress and lactation	Purple corn silage	32% dry matter extract, *ad libitum*, 12 weeks	↓ Oxidative stress (SOD); ↑ Milk production	[111]
In vivo (dairy goats) effect on oxidative stress and lactation	Commercial anthocyanin-rich purple corn pigment, Anthocyanin-rich purple corn stover silage	1 g/die, 7 days	↓ Oxidative stress (SOD, GPX2); ↑ Milk production (TNF, mRNA expression)	[112]
In vivo (dairy goats) effect on oxidative stress and lactation	Anthocyanin-rich purple corn stover silage	862 mg/kg dry matter, 6 weeks	↓ Oxidative stress (SOD, total antioxidant capacity); ↑ Milk production	[113]
In vivo (rats) effect on learning and memory capability	Blue corn tortilla	6 g/day, 28 days	↑ Learning and short-and long-term memory (Barnes’s labyrinth test)	[114]
In vivo (menopausal-model Wistar rats) neuroprotective effect	Purple waxy corn cob and pandan leaves extract (1:1, *w*/*w*)	20, 40, 80 mg/kg, 28 days	↑ Non-spatial memory (ERK1/2, neuron density); ↓ Oxidative stress status (AChE)	[115]
In vivo (menopausal-model Wistar rats) effect on cognitive function	Purple waxy corn cob and pandan leaves extract (1:1, *w*/*w*)	20, 40, 80 mg/kg, 28 days	↑ Spatial memory (ERK1/2, neuron density); ↓ Oxidative stress status (AChE)	[116]
In vitro cellular anti-cancer properties (HepG2, H-460, Hela, MCF-7, PC-3) and antioxidant effect	Blue corn and tortilla extracts	1 mg/mL	↑ Anti-proliferative effect (MTT assays); ↑ Antioxidant activity (DPPH, TBARS)	[87]
In vitro cellular anti-cancer properties (HCT-116, HT-29)	Purple and red corn extracts	0.25–10 mg/mL	↓ Angiogenesis (VEGF expression, Tie-2 marker); ↑ Apoptosis (BCL2 Associated X*BAX, B-cell lymphoma 2-Bcl-2, cytochrome c, TRAILR2/D5)	[84]
In vitro cellular anti-proliferative effect (CCA and KKU214GemR cell lines)	Anthocyanin complex (purple waxy corn and blue butterfly pea petals)	100–800 μg/mL	↑ Citotoxicity effect (FOXM1, NF-κB, Bcl-2, mitochondrial superoxide production)	[117]
In vivo (male rats) anti- orofacial allodynia effect	Anthocyanin-enriched purple corn extract (titrated 4% anthocyanin)	53 mg anthocyanins/kg body, 11 days before and 3 day after experiments	↓ Trigeminal macrophage infiltration (modulation of microglia reactivity); ↑ Von Frey Behavioral Test	[118]
In vivo (mice) skin protection	Purple corn	50% purple corn cream, 4 weeks, twice a day	↓ Photoaging (MMP-1, collagen in dermis)	[119]
In vitro (human gingival fibroblast) and in vivo (rats) wound healing	Purple waxy corn cob and *Clitoria ternatea* blue petals extract	1 or 10% *w*/*w* in niosomes	↑ in vitro permeation and wound healing	[120]
In vitro effect on cell survival (murine HL-1 cardiomyocytes) In vivo (C57BL/6J mice) cardioprotective effect	Cyanidin-3-glucoside Anthocyanin-rich B1 Pl1 hybrid seeds powder	125 mM Diet based on 29% of corn (0.21 ± 0.1 mg anthocyanin/g powder), 3 weeks before—74 days after the induction of cardiotoxicity with doxorubicin	↑ Cell viability; ↑ Animal survival; ↓ Histopathological alterations	[121]
In vivo (rats) aphrodisiac activity	Purple corn extract	25–75 mg/kg single oral treatment	Facilitates the arousal and execution of sexual behavior; ↑ Number of discharges of the ejaculatory motor patterns and total number of genital motor patterns	[122]
In vivo (rainbow trout) antioxidant effect and lipid peroxidation inhibition	Purple corn extract	5% extract feeding, twice a day, 8 weeks	↑ Antioxidant activity (gpx1 enzyme, TAC, n-3 and n-6 PUFA)	[123]
